# Hypoxia induces DOT1L in articular cartilage to protect against osteoarthritis

**DOI:** 10.1172/jci.insight.150451

**Published:** 2021-12-22

**Authors:** Astrid De Roover, Ana Escribano Núñez, Frederique M.F. Cornelis, Chahrazad Cherifi, Leire Casas-Fraile, An Sermon, Frederic Cailotto, Rik J. Lories, Silvia Monteagudo

**Affiliations:** 1 Laboratory of Tissue Homeostasis and Disease, Skeletal Biology and Engineering Research Center, Department of Development and Regeneration, KU Leuven, Leuven, Belgium.; 2 Division of Trauma Surgery, University Hospitals Leuven, Leuven, Belgium.; 3 Locomotor and Neurological Disorders Unit, Department of Development and Regeneration, KU Leuven, Leuven, Belgium.; 4 UMR 7365 CNRS — University of Lorraine, Molecular Engineering and Articular Physiopathology, Biopôle, University of Lorraine, Campus Biologie-Santé, Vandoeuvre-Les-Nancy, France.; 5 Division of Rheumatology, University Hospitals Leuven, Leuven, Belgium.

**Keywords:** Aging, Bone Biology, Cartilage, Osteoarthritis

## Abstract

Osteoarthritis is the most prevalent joint disease worldwide, and it is a leading source of pain and disability. To date, this disease lacks curative treatment, as underlying molecular mechanisms remain largely unknown. The histone methyltransferase DOT1L protects against osteoarthritis, and DOT1L-mediated H3K79 methylation is reduced in human and mouse osteoarthritic joints. Thus, restoring DOT1L function seems to be critical to preserve joint health. However, DOT1L-regulating molecules and networks remain elusive, in the joint and beyond. Here, we identified transcription factors and networks that regulate DOT1L gene expression using a potentially novel bioinformatics pipeline. Thereby, we unraveled a possibly undiscovered link between the hypoxia pathway and DOT1L. We provide evidence that hypoxia enhanced DOT1L expression and H3K79 methylation via hypoxia-inducible factor-1 **α** (HIF1A). Importantly, we demonstrate that DOT1L contributed to the protective effects of hypoxia in articular cartilage and osteoarthritis. Intra-articular treatment with a selective hypoxia mimetic in mice after surgical induction of osteoarthritis restored DOT1L function and stalled disease progression. Collectively, our data unravel a molecular mechanism that protects against osteoarthritis with hypoxia inducing DOT1L transcription in cartilage. Local treatment with a selective hypoxia mimetic in the joint restores DOT1L function and could be an attractive therapeutic strategy for osteoarthritis.

## Graphical abstract

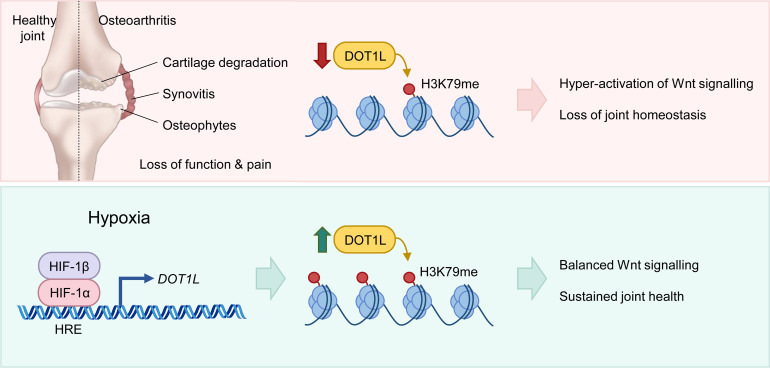

## Introduction

Osteoarthritis (OA) remains the most common chronic joint disease; it is a leading cause of disability with increasing incidence worldwide. It is characterized by progressive damage to the articular cartilage, varying degrees of synovial inflammation, subchondral bone remodeling, and osteophyte formation, leading to pain and loss of joint function (1, 2). Relevant molecular mechanisms with a role in the onset and progression of OA remain elusive. This may explain why current treatment is limited to symptom relief or joint replacement surgery and no disease-modifying therapy is available.

Restoring the histone methyltransferase disruptor of telomeric silencing 1-like (DOT1L) may be an attractive strategy for therapy. The *DOT1L* gene encodes an enzyme that methylates lysine 79 of histone H3 (H3K79) and is involved in epigenetic regulation of transcription (3–5). Previously, polymorphisms in *DOT1L* have been associated with OA (6, 7). How DOT1L affects OA remained unknown until we identified DOT1L as master protector of cartilage health (8). DOT1L activity, indicated by levels of methylated H3K79, is decreased in damaged areas from cartilage of patients with OA compared with corresponding preserved areas and non-OA cartilage. Loss of DOT1L activity in human articular chondrocytes (hACs) from healthy donors shifted their molecular signature toward an OA-like profile. In mice, intra-articular injection of a DOT1L inhibitor triggered OA. Heterozygous cartilage-specific *Dot1l*-knockout (*Dot1l*^CART-KO^) mice spontaneously developed severe OA upon aging (9). Postnatal tamoxifen-induced conditional *Dot1l*^CART-KO^ mice developed more severe posttraumatic OA upon joint injury and spontaneous OA upon aging compared with wild-type animals (9). Mechanistically, DOT1L’s protective role is accomplished via restricting signaling of Wnt, a pathway that when hyperactivated leads to OA and that is increasingly recognized as potential therapeutic target (8, 10, 11).

Factors that regulate DOT1L levels and activity in the joint remain unknown. Targeting such mechanisms to maintain or restore DOT1L function appears to be a novel therapeutic opportunity to keep an optimal balance of Wnt signaling in cartilage, preserve joint health, and inhibit progression of OA. Here, we aimed to discover DOT1L-regulating transcription factors (TFs) and networks by analyzing the human *DOT1L* promoter using a bioinformatics pipeline. We identified a potentially new mechanistic link between the hypoxia pathway and DOT1L and validated this mechanism as a therapeutic strategy to restore DOT1L function in articular cartilage and protect the joint against OA.

## Results

### A potentially novel bioinformatics pipeline identifies TFs regulating the DOT1L gene.

**Figure 1:**
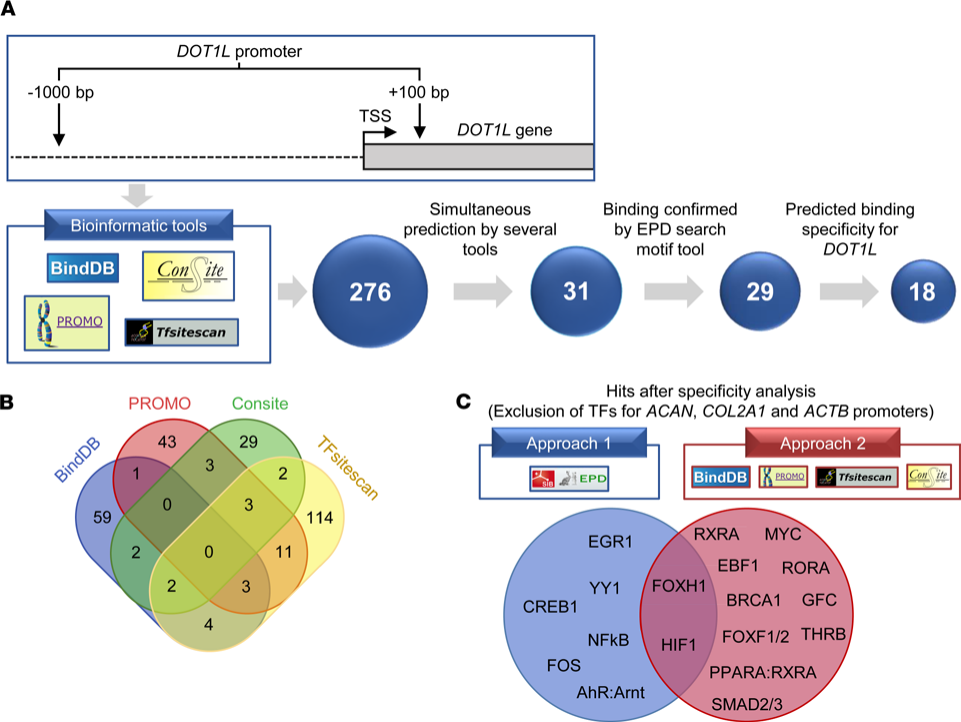
Bioinformatics pipeline identifies transcription factors regulating the *DOT1L* gene. (**A**) Overview of the bioinformatics analysis flow of the human DOT1L proximal promoter. The top part of **A** displays the *DOT1L* gene promoter region that was used for the analysis, namely –1000 bp to +100 bp relative to the transcription start site (TSS). The bottom part shows the 4 different bioinformatics web-based tools that were used and the transcription factor (TF) selection pipeline. (**B**) Venn diagram of the 276 TFs found by the 4 different tools, of which the TFs predicted by at least 2 different tools were selected for further analysis. (**C**) Overview of hits remaining after the specificity analysis. Two different approaches were used to determine whether the TFs were more specific for the *DOT1L* promoter compared with the aggrecan (*ACAN*), collagen 2a1 (*COL2A1*), and actin (*ACTB*) promoters. The diagram shows the 18 resulting TFs predicted to be more specific for *DOT1L* after exclusion of TFs by approach 1 or 2 as well as their overlap.

To identify upstream signals regulating *DOT1L* gene expression, we conducted a bioinformatics analysis of the *DOT1L* promoter, using a pipeline we designed (Figure 1A). First, the DNA sequence of the human *DOT1L* proximal promoter was obtained from the eukaryotic promoter database (EPD) (12). We analyzed this sequence with different bioinformatic tools, namely BindDB, PROMO, CONSITE, and TFsitescan (13–16). We found 276 TFs predicted to interact with the *DOT1L* promoter in silico. As these bioinformatic tools use different algorithms and TF databases, we compared the outputs. This resulted in 31 TFs simultaneously predicted by at least 2 different tools, which were selected for further analysis (Figure 1, A and B). To in silico interrogate binding of the 31 TFs to the *DOT1L* promoter, we analyzed these individually using the Search Motif from the EPD website, as this tool predicts putative TF-binding sites. This analysis corroborated putative binding of 29 TFs (Figure 1A).

Then, we assessed the potential specificity of these 29 TFs for *DOT1L*. We excluded TFs in silico predicted to bind to the promoters of genes that characterize the chondrocyte identity, namely aggrecan (*ACAN*) and collagen 2a1 *(COL2A1)*, as well as housekeeping gene actin, using two different approaches. The first used the EPD Search Motif tool to individually interrogate whether TFs predicted for *DOT1L* also bind to the promoter of the 3 mentioned control genes (Figure 1C). The second approach analyzed the promoter sequences of the 3 control genes using the same bioinformatic tools as for *DOT1L* and determined whether any of selected TFs appears in the output (Figure 1C). Combining both approaches resulted in 18 TFs that may selectively regulate *DOT1L* (Figure 1, A and C). These TFs include thyroid hormone receptor β (THRB), previously reported to increase *Dot1l* expression in tadpole intestine (17), validating our in-house analysis pipeline.

### Bioinformatics analysis unravels an undiscovered link between hypoxia and DOT1L.

**Figure 2:**
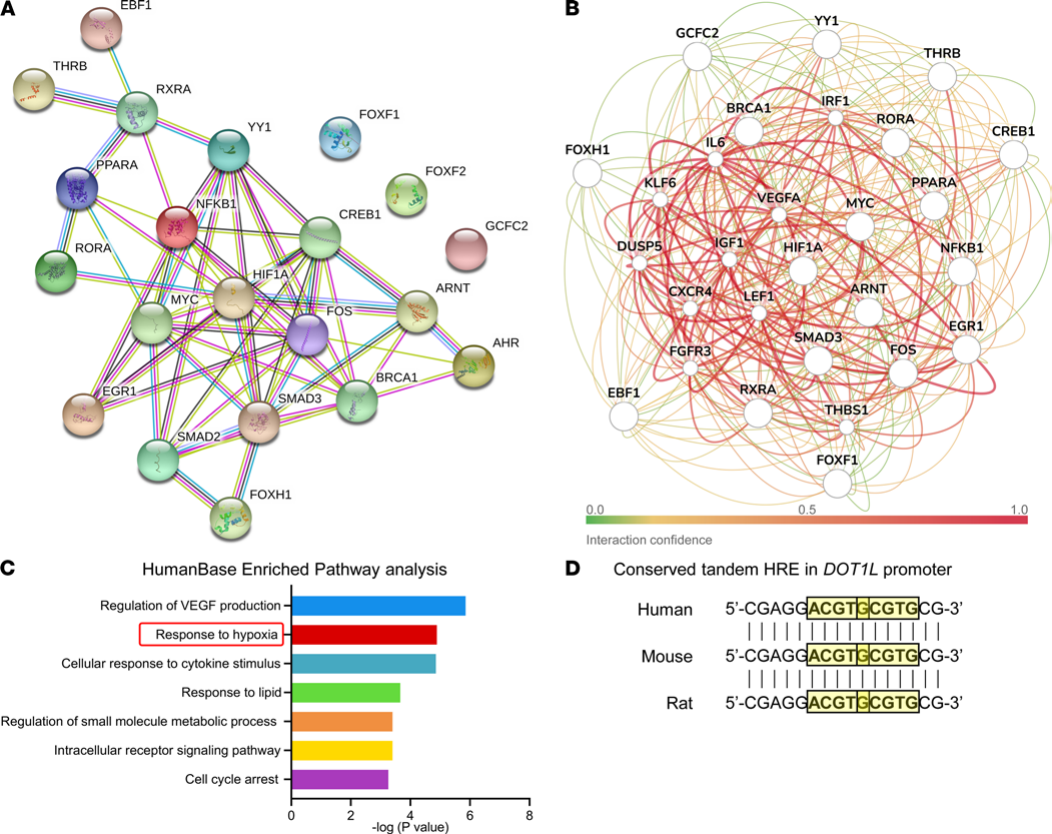
Bioinformatics analysis unravels an undiscovered link between hypoxia and *DOT1L*. (**A**) STRINGdb protein-protein network of the 18 resulting transcription factors (TFs) upon the specificity analysis. (**B** and **C**) Cartilage-specific gene network of the 18 resulting TFs upon the specificity analysis using HumanBase (GIANT) (**B**) and its pathway enrichment analysis (**C**). (**D**) Presence of tandem hypoxia-response elements (HREs) with consensus sequence 5′-(A/G)CGTG-3′ (highlighted with yellow boxes) within the human, mouse, and rat *DOT1L* gene promoters.

Next, we explored interactions and regulatory networks of the obtained TFs. To this end, we used STRINGdb (18), a database of known and predicted protein-protein interactions (Figure 2A). We also used HumanBase (GIANT) to build a cartilage-specific network (Figure 2B) (19). In the regulatory networks obtained, there was a prominent node around hypoxia-inducible factor-1 α (HIF1A) (Figure 2, A and B), and the hypoxia pathway was enriched (Figure 2C). In our in silico specificity assessment (Figure 1C), HIF1A was found to be specific for *DOT1L* by both approaches.

HIFs mediate the transcriptional response to low oxygen tension or hypoxia. They are heterodimeric TFs consisting of an unstable oxygen-sensitive α subunit and a stable β subunit (20). In the presence of oxygen, HIF-α is degraded. Under hypoxia, HIF-α is stabilized and dimerizes with β subunit. The heterodimers bind to hypoxia-response elements (HREs) in the genome, regulating gene expression.

Adult articular cartilage is avascular and physiologically in a hypoxic state (21). However, in OA, the hypoxic nature of cartilage is disrupted (22, 23). Mammals have 3 isoforms of the α subunit: HIF1A, HIF2A, and HIF3A (24). Within human cartilage, the isoforms HIF1A and HIF2A are the main mediators of transcriptional responses to hypoxia (25). HIF1A promotes cartilage homeostasis (21, 23, 26). In contrast, HIF2A is associated with chondrocyte hypertrophy and a catabolic response (27, 28).

HIFs can bind consensus sequence 5′-(A/G)CGTG-3′ within the HRE but show differences in target gene specificity (20, 24, 25, 27). We identified HREs with consensus sequence 5′-(A/G)CGTG-3′ in the *DOT1L* promoter, including overlapping tandem HREs (Figure 2D and Supplemental Figure 1; supplemental material available online with this article; https://doi.org/10.1172/jci.insight.150451DS1). Two tandem HREs result in a stronger transcriptional response compared with one HRE (29). Alignment of the human, mouse, and rat *DOT1L* promoters revealed that the overlapping tandem HREs and surrounding region were conserved, highlighting the possible relevance of this regulatory motif (Supplemental Figure 2).

### Hypoxia increases DOT1L expression and H3K79 methylation in hACs.

**Figure 3:**
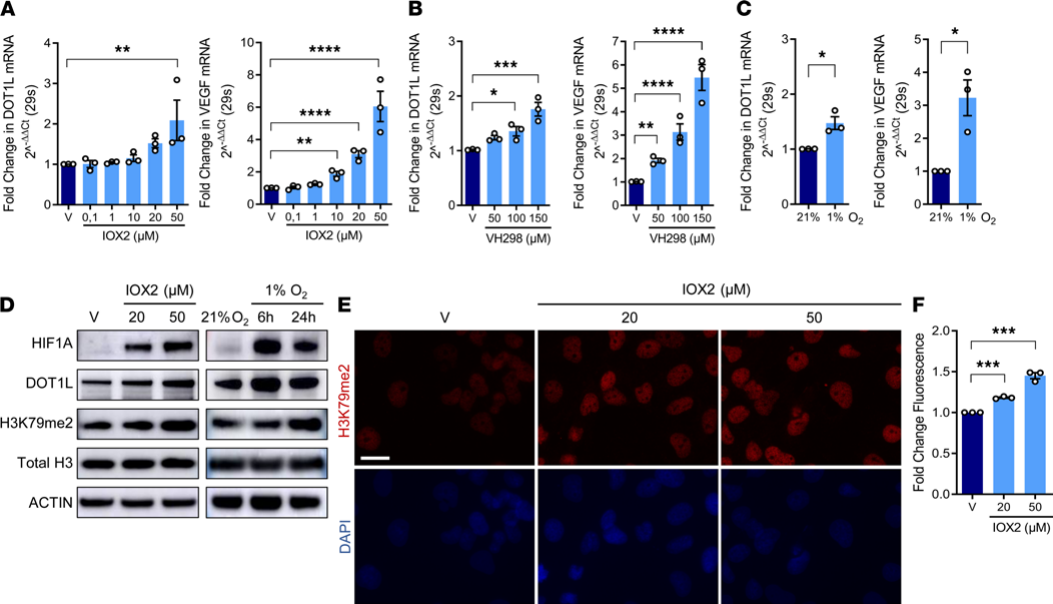
Hypoxia increases *DOT1L* and H3K79 methylation in human articular chondrocytes. (**A** and **B**) Real-time PCR for DOT1L and VEGF in C28/I2 cells treated with IOX2, VH298, or vehicle (V) for 72 hours (C28/I2, *P < 0.05, **P < 0.01, ***P < 0.001, ****P < 0.0001 in **A** and **B**, Holm corrected for 16 and 6 tests by generalized least squares model). (**C**) Real-time PCR in normoxic (21% O_2_) or hypoxic (1% O_2_) conditions for 6 hours (*n* = 3, *P < 0.05 by Welch-corrected *t* test). (**D**) Immunoblot of hypoxia-inducible factor-1 α (HIF1A), DOT1L, and H3K79 methylation after IOX2 treatment for 96 hours and in response to hypoxia. Images are representative of 2 independent experiments. (**E** and **F**) H3K79 methylation by immunofluorescence (red) and DAPI staining (blue) in C28/I2 cells after 72 hours. Images are representative of 3 independent experiments with technical duplicates. Scale bar: 50 μm. Fluorescence intensity per cell relative to V (*n* = 20 images per condition for each experiment; *n* = 3, ***P < 0.001, Holm corrected for 3 tests by generalized least squares model by generalized least squares model).

To investigate whether hypoxia regulates the *DOT1L* gene in articular cartilage, we studied effects of hypoxia mimetics or low oxygen levels on C28/I2 cells, a hAC cell line (30). We used quantitative PCR to determine expression of *DOT1L* and positive control *VEGF*, a well-established hypoxia target gene. First, we treated C28/I2 cells with 2 pharmacological hypoxia mimetics. Treatment with IOX2 increased *DOT1L* expression in a concentration-dependent manner (pseudo-*R*^2^ = 0.73, *P* < 0.0001) (Figure 3A). Treatment with VH298 similarly led to increased *DOT1L* expression (pseudo-*R*^2^ = 0.85, *P* < 0.0001) (Figure 3B). Likewise, culturing C28/I2 cells in a hypoxia chamber promoted *DOT1L* expression (1.46-fold increase [95% CI, 1.05–2.02] *P* = 0.0385) (Figure 3C). As expected, *VEGF* expression was increased in all experimental conditions (pseudo-*R*^2^ = 0.92, *P* < 0.0001; *R*^2^ = 0.97, *P* < 0.0001; 3.12-fold increase [95% CI, 1.42–6.84], *P* = 0.0248; respectively) (Figure 3, A–C).

Then, we interrogated whether hypoxia also leads to increased DOT1L protein and H3K79 methylation, using Western blot. Both IOX2 and incubation in a hypoxia chamber stabilized HIF1A and increased DOT1L protein and H3K79 methylation in hACs (Figure 3D). Immunofluorescence further demonstrated increased DOT1L-mediated H3K79 methylation upon IOX2 treatment (pseudo-*R*^2^ = 0.97, *P* < 0.0001) (Figure 3, E and F). Altogether, these data indicate that hypoxia enhances DOT1L expression and H3K79 methylation in chondrocytes.

### Hypoxia-mediated induction of DOT1L depends on HIF1A but not HIF2A.

**Figure 4:**
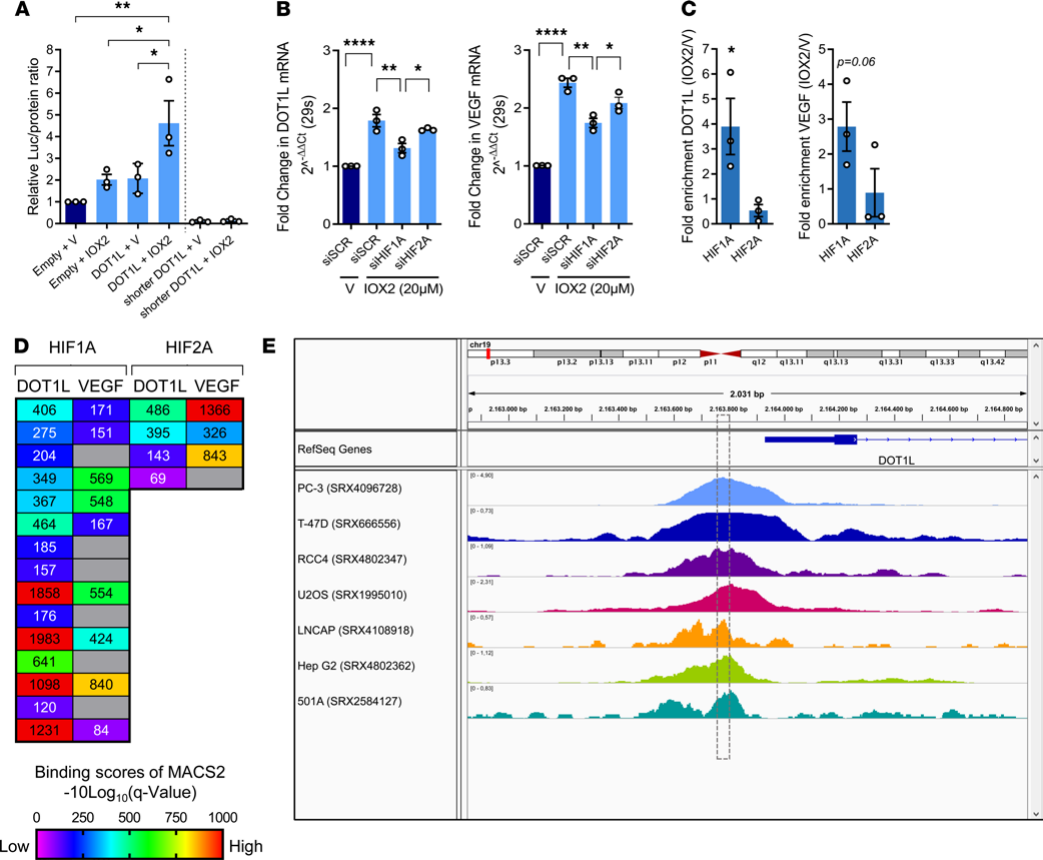
Hypoxia-mediated induction of *DOT1L* depends on HIF1A but not HIF2A. (**A**) Luciferase assay in C28/I2 cells transfected with empty plasmid, full *DOT1L* promoter reporter, or negative control shorter *DOT1L*-promoter reporter, without conserved overlapping tandem HREs, upon treatment with IOX2 (20μM) for 72 hours, normalized to total protein relative to empty plasmid and vehicle (V) (*n* = 3, *P < 0.05, **P < 0.01, Šidák corrected for 6 tests in 2-way ANOVA). (**B**) Real-time PCR with siRNA-mediated silencing of *HIF1A*, *HIF2A*, or scrambled control (siSCR) (*n* = 3, *P < 0.05, **P < 0.01, ****P < 0.0001, Šidák corrected for 6 tests in 1-way ANOVA). (**C**) ChIP quantitative PCR (ChIP-qPCR) for HIF1A and HIF2A binding to *DOT1L* and *VEGF* promoters in cells treated with IOX2 (20 μM) for 72 hours (*n* = 3, *P < 0.05 by 1-sided *t* test). (**D**) MACS2-binding scores around *DOT1L* and *VEGF* transcription start site (TSS) of publicly available HIF1A and HIF2A ChIP-Seq data (ChIP-Atlas database). (**E**) Visualization of HIF1A ChIP-Seq in various cells around the *DOT1L* TSS. Box indicates overlapping HREs. ChIP-Atlas data mapped to reference human genome (hg19) using Integrative Genomics Viewer (IGV). Data are shown as the mean ± SEM.

Next, we explored the molecular mechanism via which hypoxia induces *DOT1L* expression. First, we evaluated functionality of the conserved overlapping tandem HREs present in the *DOT1L* promoter using a luciferase assay. To this end, the full human *DOT1L* promoter (–1000 bp to +91 bp relative from the transcription start site [TSS]) was synthesized and cloned into the pGL3-basic vector upstream of a reporter luciferase gene. A shorter *DOT1L* promoter (–412 bp to +91 bp relative from TSS) in which the conserved overlapping tandem HREs were absent was used as a negative control (Supplemental Figure 3). These plasmids were transfected into C28/I2 cells followed by hypoxia mimetic IOX2 treatment. Luciferase activity was increased upon IOX2 stimulation in the full *DOT1L* promoter construct (2.21-fold [95% CI, 1.01–4.80], *P* = 0.0460) compared with control (Figure 4A). With the shorter promoter no reporter activity was detected. Thus, the conserved overlapping tandem HREs are functional and required for hypoxia-induced promoter activity.

Further, we interrogated the roles of HIF1A and HIF2A in *DOT1L* transcription. Silencing of *HIF1A* blocked IOX2-mediated induction of *DOT1L* expression, while silencing of *HIF2A* had no effects in C28/I2 cells treated with hypoxia mimetic IOX2 (1.36-fold higher for siSCR vs. siHIF1A [95% CI, 1.10–1.67], *P* = 0.0055 for *DOT1L*, 1.4-fold higher for *VEGF* [95% CI, 1.17–1.67], *P* = 0.0011) (Figure 4B and Supplemental Figure 4). ChIP-qPCR in C28/I2 cells treated with IOX2 demonstrated that HIF1A, but not HIF2A, localized at the *DOT1L* gene promoter (3.59-fold [95% CI, 1.07–12.09], *P* = 0.045 for HIF1A) (Figure 4C). To further confirm this molecular mechanism, we interrogated *DOT1L* as a potential target gene of HIF1A and HIF2A using ChIP-Atlas (31), an integrative and comprehensive data-mining suite of public ChIP-Seq data. This analysis revealed relatively higher MACS2 scores for HIF1A compared with HIF2A, indicating higher binding to *DOT1L* (Figure 4D). Binding of HIF1A to the *DOT1L* promoter was found around the area of the overlapping HREs in multiple ChIP-Atlas data sets (Figure 4E). Collectively, these data demonstrate that hypoxia directly regulates *DOT1L* expression via HIF1A.

### DOT1L contributes to the protective effects of hypoxia in hACs.

**Figure 5:**
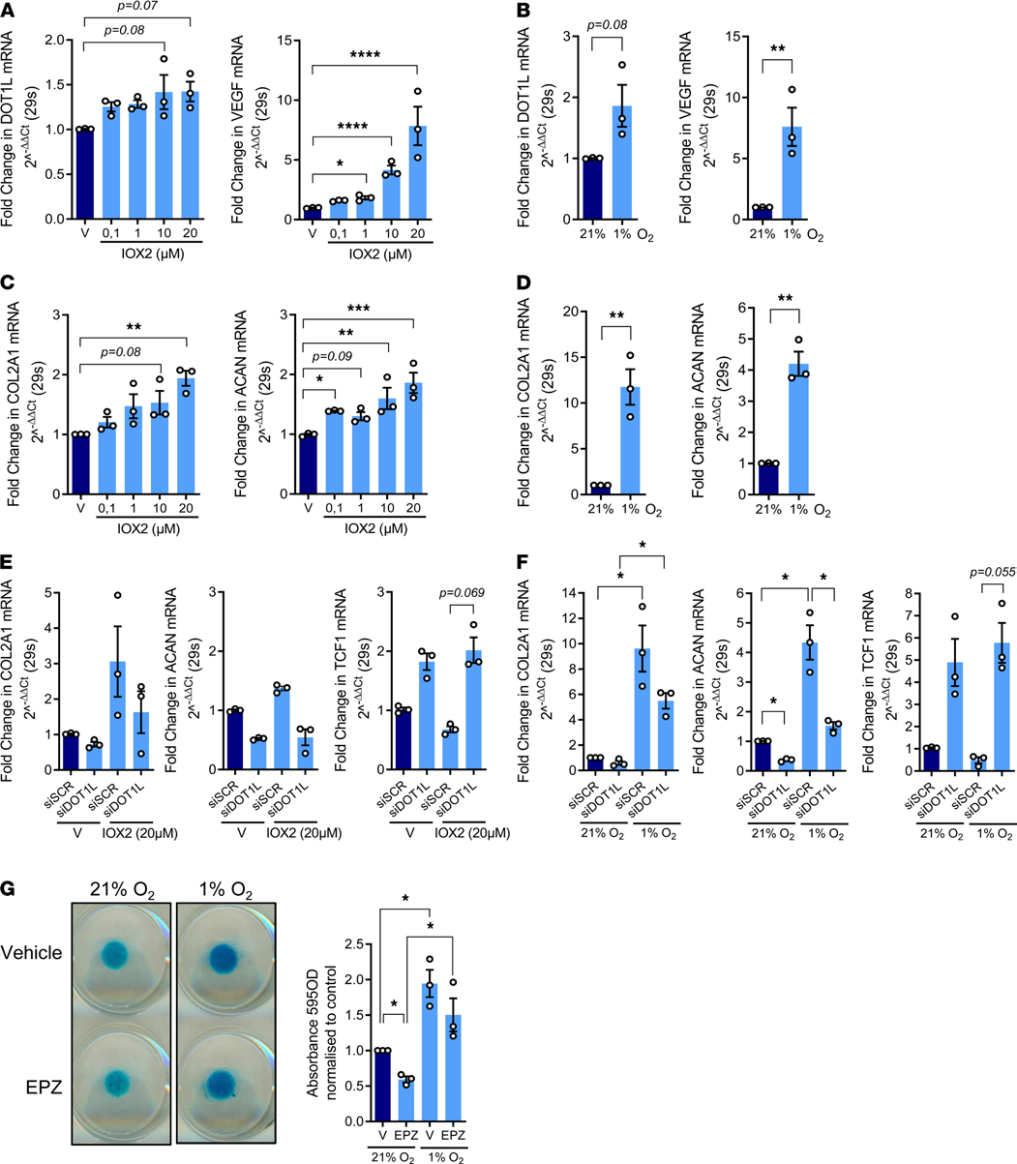
DOT1L contributes to the protective effects of hypoxia in human articular chondrocytes. (**A**) Real-time PCR analysis of *DOT1L* and *VEGF* in primary human articular chondrocytes (hACs) after treatment with hypoxia mimetic IOX2 or vehicle (V) at the indicated concentrations for 72 hours (*n* = 3, *P < 0.05, ****P < 0.0001, Holm corrected for 10 tests by generalized least squares model). (**B**) Real-time PCR analysis of *DOT1L* and *VEGF* in primary hACs cultured in normoxic (21% O_2_) or hypoxic (1% O_2_) conditions for 14 days (*n* = 3, **P < 0.01 by paired *t* test). (**C**) Real-time PCR analysis of *COL2A1* and *ACAN* in primary hACs after treatment with IOX2 at the indicated concentrations or V for 72 hours (*n* = 3, *P < 0.05, **P < 0.01, ****P < 0.0001, Holm corrected for 10 tests by generalized least squares model). (**D**) Real-time PCR analysis of *COL2A1* and *ACAN* in primary hACs cultured in normoxic (21%O_2_) or hypoxic (1%O_2_) conditions for 14 days (*n* = 3, **P < 0.01 by paired *t* test). (**E**) Real-time PCR analysis of *COL2A1*, *ACAN*, and *TCF1* in primary hACs after treatment with hypoxia mimetic IOX2 (20 μM) or V and siRNA-mediated silencing of *DOT1L* (siDOT1L) or scrambled control (siSCR) for 72 hours (*n* = 3, *P* < 0.05, Šidák corrected for 6 tests in 2-way ANOVA). (**F**) Real-time PCR analysis of *COL2A1*, *ACAN*, and *TCF1* in primary hACs cultured in normoxic (21%O_2_) or hypoxic (1%O_2_) conditions for 14 days and siRNA-mediated silencing of *DOT1L* or siSCR (*n* = 3, *P < 0.05, Šidák corrected for 6 tests in 2-way ANOVA). (**G**) Alcian blue staining of primary hAC micromasses cultured in normoxic (21%O_2_) or hypoxic (1%O_2_) conditions treated with V or DOT1L inhibitor EPZ-5676 (EPZ) for 14 days. Images are representative of 3 independent experiments with technical triplicates. Quantification of staining relative to V in normoxic conditions was determined by colorimetry at 595 nm (*n* = 3, *P < 0.05 Šidák corrected for 6 tests in 2-way ANOVA). Data are shown as the mean ± SEM.

To translationally validate these findings, we assessed effects of hypoxia on primary hACs. IOX2 treatment induced *DOT1L* expression in these cells (*R*^2^ = 0.27, *P* = 0.040), whereas culture in hypoxic conditions suggested a similar trend (1.80-fold increase [95% CI, 0.83–3.88] *P* = 0.081) (Figure 5, A and B). Expression of positive control *VEGF* was also induced (*R*^2^ = 0.95, *P* < 0.0001; 7.27-fold increase [95% CI, 3.17–16.68], *P* = 0.0093; respectively] (Figure 5, A and B). Then, we verified that a hypoxic environment is beneficial for the molecular phenotype of the articular chondrocyte. Hypoxia mimetic IOX2 increased expression of *COL2A1* and *ACAN* (*R*^2^ = 0.56, *P* = 0.001; *R*^2^ = 0.66, *P* = 0.0006; respectively) (Figure 5C). In addition, culturing primary hACs in a hypoxia chamber induced healthy chondrocyte markers (11.36-fold increase for *COL2A1* [95% CI, 5.53–23.36], *P* = 0.0047; 4.14-fold for *ACAN* [95% CI, 2.88–5.95] *P* = 0.0035) (Figure 5D).

Our data demonstrate that hypoxia induces *DOT1L* expression and has protective effects in primary hACs. A key question is whether DOT1L contributes to hypoxia’s protective effects. To answer this, we evaluated effects of IOX2 or a hypoxia chamber in the absence of DOT1L in primary hACs. Whereas IOX2 treatment in control conditions resulted in similar changes in *COL2A1* and *ACAN* expression, as seen above (Figure 5, C and E), and silencing of *DOT1L* using siRNA had a global effect on the expression of *ACAN* in the ANOVA analysis model (*P* = 0.0365 for main effect), no clear differences were found in the post hoc pair-wise comparisons. In contrast, culturing hACs in a hypoxia chamber increased *COL2A1* expression (*P* = 0.0044 for main effect), but *DOT1L* levels had no significant effect (*P* = 0.0693 for main effect). For *ACAN*, hypoxia increased (*P* = 0.0063 for main effect) and silencing of *DOT1L* decreased (*P* = 0.0149) the gene expression levels. Post hoc pair-wise analyses confirmed that silencing of *DOT1L* negatively affected *ACAN* expression under both culture type conditions (2.78-fold decrease in normoxia [95% CI, 1.02–7.52], *P* = 0.0478; 2.82-fold decrease in hypoxia [95% CI, 1.04–7.63], *P* = 0.0465). We earlier demonstrated that DOT1L’s protective role is exerted via limiting Wnt signaling (8, 9). Therefore, we assessed Wnt signaling activity in IOX2-treated as well as hypoxia-incubated hACs by measuring expression of *TCF1*, a direct Wnt target gene epigenetically regulated by DOT1L (8). Silencing of *DOT1L* increased *TCF1* expression (*P* = 0.0076 for main effect in the hypoxia chamber experiments, Figure 5F; *P* = 0.010 in the IOX2 experiments, Figure 5E and Supplemental Figure 5). Post hoc pair-wise analyses were likely underpowered but suggested that the effect was most present under hypoxia or hypoxia mimicked by IOX2 treatment (13.14-fold increase in the hypoxia chamber [95% CI, 0.87–197.15], *P* = 0.055; 2.95-fold increase after IOX2 treatment [95% CI, 0.81–10.66], *P* = 0.069). We further used 3-dimensional micromass cultures of primary hACs. Culturing these micromasses under hypoxia increased glycosaminoglycan content, as determined by Alcian blue staining (*P* = 0.0127 for main effect in the ANOVA model), while DOT1L inhibition with EPZ-5676 (EPZ) partially blocked this increase (*P* = 0.0212 for main effect) (Figure 5G). Post hoc pair-wise analyses confirmed that inhibition of DOT1L negatively affected glycosaminoglycan content in normoxia cultures (1.69-fold decrease [95% CI, 1.04–2.75], *P* = 0.0426), whereas hypoxia increased glycosaminoglycan content in both control and EPZ-treated micromasses (1.93-fold increase in vehicle treated [95% CI, 1.18–3.12], *P* = 0.0278 and 2.48-fold increase in EPZ treated [95% CI, 1.53–4.04], *P* = 0.0145). Taken together, these in vitro data confirm that low oxygen levels support cartilage health and that DOT1L may contribute to this.

### Intra-articular treatment with IOX2 halts OA in mice and restores DOT1L in articular cartilage.

**Figure 6:**
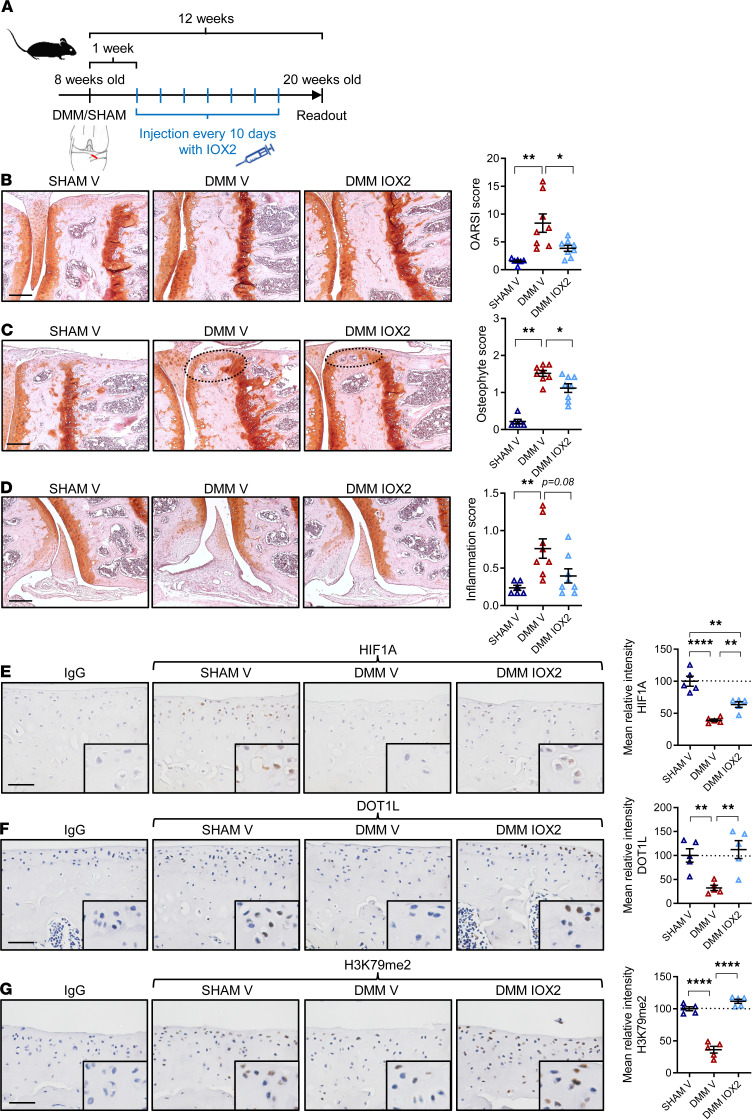
Intra-articular injection of IOX2 halts OA in mice and restores DOT1L in articular cartilage. (**A**) Time course of intra-articular injections of IOX2 or vehicle (V) in DMM or sham-operated wild-type mice. (**B**) Frontal hematoxylin-safraninO staining of the medial tibia and femur and quantification of articular cartilage damage at the 4 quadrants, evaluated by OARSI score. (**C**) Frontal hematoxylin-safraninO staining of the medial tibia and femur and quantification of osteophytes at the medial tibia and femur. (**D**) Frontal hematoxylin-safraninO staining of the lateral synovium and quantification of inflammation. (**B–D**) Scale bar: 200 μm. Data are presented as individual data points (*n* = 6 [SHAM V] and *n* = 8 [DMM V and DMM IOX2], *P < 0.05, **P < 0.01, Holm-Bonferroni–corrected for 3 tests in Kruskal-Wallis test). (**E–G**) Immunohistochemical detection of HIF1A, DOT1L, and H3K79me2 in the articular cartilage of wild-type mice treated with IOX2 or V upon OA triggered by DMM surgery compared with sham-operated mice. Scale bar: 50 μm (*n* = 5 per group, **P < 0.01, ****P < 0.0001, Šidák corrected for 3 tests in 1-way ANOVA). Data are shown as the mean ± SEM.

We investigated therapeutic implications of our findings for OA. We previously showed that DOT1L and H3K79 methylation are reduced in human and mouse OA cartilage compared with non-OA cartilage (8, 9). In addition, HIF1A is reduced in OA cartilage (22, 23, 27). Yet, to our knowledge, local administration of selective hypoxia mimetics has not been evaluated in a clinically relevant mouse model of OA. We investigated effects of a hypoxia mimetic on OA using the destabilization of the medial meniscus (DMM) mouse disease model (32, 33). Before initiating in vivo treatments, we corroborated that DMM-operated mice showed a concomitant downregulation in DOT1L and HIF1A proteins in articular cartilage compared with sham-operated controls (Supplemental Figure 6). Upon these observations, we proceeded with the in vivo pharmacological intervention. Starting 1 week after DMM surgery, mice were intra-articularly injected with IOX2 every 10 days, and knee joints were collected 12 weeks after surgery (Figure 6A). Histological analysis showed that IOX2 treatment reduced cartilage damage (*r* = 0.578 [95% CI, 0.16-0.82], *P* = 0.021 for IOX2 treated versus vehicle in DMM) and osteophyte formation (*r* = 0.567 [95% CI, 0.13-0.82], *P* = 0.027) and possible synovial inflammation (*r* = 0.527 [95% CI, 0.10–0.82], *P* = 0.08) upon DMM (Figure 6, B–D). Immunohistochemistry showed that HIF1A, DOT1L, and H3K79 methylation levels were decreased in the DMM model compared with sham controls (difference of means in relative intensity, 2.54 [95% CI, 1.94–3.33], *P* < 0.001; 3.13 [95% CI, 1.57–6.24], *P* < 0.019; 2.90 [95% CI, 1.99–4.24], *P* < 0.001; respectively]. IOX2 treatment effectively rescued HIF1A (difference of means, 1.62 [95% CI, 1.24–2.10], *P* = 0.001) and increased DOT1L (difference of means, 3.41 [95% CI, 1.75–6.63], *P* = 0.001) and H3K79 methylation (difference of means, 3.24 [95% CI, 2.25–4.67], *P* < 0.0001) after DMM (Figure 6, E–G). These in vivo data indicate that intra-articular treatment with a selective hypoxia mimetic restores DOT1L and H3K79 methylation and protects against OA.

## Discussion

This study provides evidence that restoring hypoxia in the joint could be an attractive therapeutic strategy for OA, because it rescues DOT1L activity in cartilage. Despite DOT1L’s fundamental role in diverse biological processes, to date little is known about how DOT1L expression and activity are regulated (34, 35). We identified potential TFs that regulate the *DOT1L* gene using bioinformatics analysis. Several applications developed to predict regulatory elements have poor predictive specificity (36). Our pipeline that includes and compares multiple available online tools could effectively identify regulators of the *DOT1L* gene. Hence, our pipeline design may be used to identify regulators for any gene of interest.

The avascular cartilage is in a permanent hypoxic state throughout life. Evolutionarily, articular chondrocytes are well adapted to hypoxia and low oxygen levels maintain cartilage homeostasis (37–39). Our data indicate that hypoxia is disrupted in OA, in agreement with recent studies demonstrating increased oxygen concentrations in OA cartilage (22, 23). OA-associated cartilage damage may allow deeper synovial fluid penetration and, as a consequence, more oxygen supply to the cartilage. Another hypothesis suggests that OA synovial membrane inflammation and hypervascularity may alter the oxygen diffusion characteristics, resulting in higher oxygen concentration in synovial fluid and thus in cartilage.

Within human cartilage, HIF1A and HIF2A are the main mediators of the response to hypoxia. HIF1A has been reported to promote cartilage homeostasis in several ways. For instance, HIF1A increases expression of anabolic chondrogenic genes, such as *COL2A1* and *ACAN* (37). In addition, HIF1A suppresses catabolic proteins, such as *MMP-13* and *NFkB* (22, 37). In contrast, the role of HIF2A is still controversial and mainly associated with chondrocyte hypertrophy and a catabolic response (27, 28). Our present data demonstrating that DOT1L is regulated by HIF1A and not by HIF2A are in line with HIF1A’s established protective role.

Hypoxia promotes chondrogenic matrix genes while suppressing catabolic enzymes (39); however, underlying mechanisms remain incompletely defined. Duval et al. demonstrated that hypoxia induces chondrogenesis in mesenchymal stem cells by HIF1A binding to the *SOX9* promoter, subsequently increasing *COL2A1* expression (40). Bouaziz et al. reported that HIF1A can interact with β-catenin thereby reducing the binding of TCF4 to Wnt target gene promoters (23). Thus, we currently lack a complete understanding of oxygen-sensitive pathways and the response to hypoxia, in particular in articular cartilage. We revealed that DOT1L contributes to the protective effects of hypoxia. Silencing or inhibiting *DOT1L* reduced beneficial effects of hypoxia on primary hACs in monolayer and 3-dimensional cultures. Considering DOT1L’s role in Wnt signaling regulation, we assessed effects on Wnt activity, demonstrating that hypoxia limits Wnt signaling. This is in line with the findings of Bouaziz et al. (23). However, we also demonstrated that this effect on Wnt is impaired upon *DOT1L* silencing.

DOT1L plays a role in many biological functions, such as cell cycle regulation, DNA damage response, hematopoiesis, cardiac function, and more (4, 41, 42). Therefore, it is very probable that DOT1L is regulated by complex mechanisms. For instance, THRB has previously been reported to increase *Dot1l* expression in tadpole intestine (17). Here, we identified HIF1A as a *DOT1L* regulator; however, we acknowledge that HIF1A is not the only regulator.

We identified a regulatory motif in the *DOT1L* promoter consisting of 2 overlapping HRE tandem repeats, conserved among species. Due to steric hindrance, it is unlikely that both HREs are functional. Yet, Fukasawa et al. previously demonstrated that 2 tandem HREs result in a stronger transcriptional response compared with only 1 (29). Nevertheless, the presence of a motif does not necessarily mean that the TF will bind. Our luciferase assay results demonstrate the functionality of the overlapping tandem HREs present in the *DOT1L* promoter.

As mentioned, hypoxia increases healthy chondrocyte markers and reduces catabolic markers in normal and OA chondrocytes in vitro (39). Yet, to date, there are no reports about successful in vivo pharmacological interventions to locally restore hypoxia in OA joints. To our knowledge, only two studies investigated whether stabilization of HIF1A could prevent OA (43, 44). Both studies used dimethyloxalylglycine (DMOG), a 2-oxoglutarate (2-OG) analog that acts as a broad spectrum inhibitor against all 2-OG–dependent dioxygenases. Notably, 2-OG–dependent dioxygenases have multiple roles in cell biology, participating in oxygen sensing, lipid metabolism, collagen and carnitine biosynthesis, and histone demethylation (45–49). Gelse et al. injected DMOG intra-articularly in knees of 8-week-old STR/ort mice (43). This treatment did not ameliorate spontaneous OA, which was explained by the lack of specificity of DMOG, which interferes with collagen biosynthesis, resulting in reduced *COL2A1* expression, and induces catabolic cytokines (43). Hu et al. performed intraperitoneal injections of DMOG in 10-week-old DMM mice every day (44). Although this approach seemed to reduce cartilage damage, an important point of attention that would limit its clinical application is that such systemic approach using a broad spectrum inhibitor (DMOG) would trigger a systemic inhibition of 2-OG–dependent dioxygenases. Therefore, this setup could be regarded as not hypoxia or tissue selective. Here, intra-articular administration of IOX2, a selective hypoxia mimetic, was able to halt OA in a relevant mouse OA model.

DOT1L is the main H3K79 methyltransferase (42, 50, 51). Only one report has stated that response element II–binding protein (REIIBP) may also methylate H3K79 (52). However, these data have not yet been further corroborated or replicated. Importantly, the discovery that hypoxia controls the transcription of the *DOT1L* gene might have implications in diseases beyond OA. Of note, our ChIP-Atlas analysis showed that HIF1A binds to the *DOT1L* promoter in several cell types. This might indicate that the regulatory mechanism identified here could indeed play a role in organs and tissues beyond cartilage. Based on literature, hypoxia and DOT1L are important in several coinciding processes throughout the human body, thereby suggesting that a hypoxia-mediated regulation of DOT1L could be involved. For example, both hypoxia and DOT1L have been reported to be essential during early erythropoiesis (53, 54). In addition, hypoxia and DOT1L are both linked to the immune response and to kidney injury (55–60). In cancer, hypoxia and DOT1L also play important roles (56, 61–63).

In conclusion, our study identifies that hypoxia regulates the expression of *DOT1L* via HIF1A. We demonstrate that DOT1L contributes to the protective effects of hypoxia on OA. Translationally, local treatment with a selective hypoxia mimetic halts disease progression in the DMM mouse OA model, demonstrating that targeting hypoxia could be an attractive therapeutic strategy for OA.

## Methods

### Materials.

The hypoxia mimetics IOX2 and VH298 were purchased from MilliporeSigma and Tocris, respectively.

### Bioinformatics analysis.

The *DOT1L* proximal promoter sequence 1000 bp upstream and 100 bp downstream relative to the TSS was obtained from the online tool EPD (https://epd.epfl.ch//index.php) (12). This DNA sequence was analyzed using online freely available web-based tools to predict TFs, namely CONSITE (http://consite.genereg.net) (15), TFsitescan (http://www.ifti.org/cgi-bin/ifti/Tfsitescan.pl) (16), BindDB (http://bind-db.huji.ac.il) (13), and PROMO (http://alggen.lsi.upc.es/cgi-bin/promo_v3/promo/promoinit.cgi?dirDB=TF_8.3) (14). We compared the outputs from the different online tools. Only TFs that were predicted by at least 2 different tools were selected for further analysis. Next, we used the EPD Search Motif tool, which uses the JASPAR database, to confirm predicted binding to the *DOT1L* proximal promoter. Potential specificity for *DOT1L* was in silico interrogated by assessing the binding of these TFs to the promoters of aggrecan, collagen 2a1, and actin using two different approaches. The first approach consisted of using the EPD Search Motif tool to individually interrogate whether the selected TFs predicted to bind to the *DOT1L* gene also bind to the promoter of the control genes mentioned above. The second approach consisted of analyzing the promoter sequences of the control genes using CONSITE, TFsitescan, BindDB, and PROMO and determining whether any of the selected TFs appears in the output. Finally, STRINGdb (https://string-db.org) (18) and HumanBase (https://hb.flatironinstitute.org) (19) were used to explore protein-protein interactions and cartilage-specific regulatory networks of the predicted TFs, respectively.

### Cell culture.

Human immortalized articular chondrocyte C28/I2 cells were purchased from Merck Millipore and cultured in DMEM/F12 (Gibco) containing 10% FBS (Gibco), 1% (vol/vol) antibiotic/antimycotic (Gibco), and 1% L-glutamine (Gibco) in a humidified atmosphere at 37°C and 5 % CO_2_. For the isolation of primary hACs, cartilage was first dissected from the joint surface, rinsed with PBS, and cut into small pieces. The cartilage pieces were incubated with 2 mg/ml pronase solution (Roche) for 90 minutes at 37°C and digested overnight at 37°C in 1.5 mg/ml collagenase B solution (Roche). Then, the preparation was filtered through a 70 μM strainer and cells were plated in culture flasks and cultured in a humidified atmosphere at 37°C and 5% CO_2_. Culture medium consisted of DMEM/F12 (Gibco), 10% FBS (Gibco), 1% (vol/vol) antibiotic/antimycotic (Gibco), and 1% L-glutamine (Gibco).

### Small interfering RNA transfection.

Cells were transfected with Lipofectamine RNAiMAX (Invitrogen) as transfection reagent, together with nontargeting siGENOME siRNA (siSCR) or siGENOME siRNA against DOT1L, HIF1A or HIF2A (Dharmacon) following the protocols provided by the manufacturer.

### Quantitative PCR.

Total RNA was extracted using the Nucleospin RNA II kit (Macherey-Nagel). cDNA was synthesized using the RevertAidHminus First Strand cDNA synthesis kit (Thermo Fisher Scientific) according to the manufacturers’ guidelines. Quantitative PCR analyses were carried out as described previously using Maxima SYBRgreen qPCR master mix system (Thermo Fisher Scientific) (8). Gene expression was calculated following normalization to housekeeping gene S29 mRNA levels using the comparative Ct (cycle threshold) method. The following PCR conditions were used: incubation for 10 minutes at 95°C followed by 40 amplification cycles of 15 seconds of denaturation at 95°C followed by 45 seconds of annealing-elongation at 60°C. Melting curve analysis was performed to determine the specificity of the PCR. Primers used for qPCR analysis are listed in Supplemental Table 1.

### Cell lysis and Western blotting.

Cells were lysed in IP Lysis/Wash buffer (Thermo Fisher Scientific) supplemented with 5% (vol/vol) Protease Mixture Inhibitor (MilliporeSigma) and 1 mM phenylmethanesulfonyl (MilliporeSigma). After two homogenization cycles (7 s) with an ultrasonic cell disruptor (Microson; Misonix), total cell lysates were centrifuged at 18,000*g* for 10 minutes. The supernatant containing proteins was collected and the protein concentration was determined by Pierce BCA Protein Assay Kit (Thermo Fisher Scientific). Immunoblotting analysis was carried out as previously described (8). Antibodies against Actin (MilliporeSigma, A2066; dilution 1:4000), DOT1L (Cell Signaling, 77087; dilution 1:1000), HIF1A (Abcam, ab82832; dilution 1:1000), total H3 (Abcam, ab1791; dilution 1:10,000), and H3K79me2 (Abcam, ab3594; dilution 1:1000) were used following the manufacturer’s instructions. The blotting signals were detected using the SuperSignalWest Femto Maximum Sensitivity Substrate system (Thermo Fisher Scientific).

### ChIP analysis.

ChIP assays were performed using the Agarose ChIP kit (Thermo Fisher Scientific) according to the manufacturer’s recommendations. Briefly, cell samples were cross-linked with 1% formaldehyde for 10 minutes. This reaction was stopped by adding glycine to a 125 mM final concentration. The fixed cells were lysed and the chromatin was fragmented by nuclease digestion. Further, the sheared chromatin was incubated with antibodies against HIF1A (Abcam, ab1; dilution 1:50) and HIF2A (Abcam, ab199; dilution 1:50) and recovered by binding to protein A/G agarose. Eluted DNA fragments were used directly for qPCR. Primers used for ChIP-qPCR analysis are listed in Supplemental Table 2.

Bioinformatics in silico ChIP analysis was performed using the ChIP-Atlas (https://chip-atlas.org) (31), an integrative and comprehensive data-mining suite of public ChIP-Seq data. The feature Target Genes was used to predict target genes bound by the given TFs HIF1A and HIF2A. From these results, the individual ChIP-Seq experiments that showed binding to *DOT1L* were selected for further analysis. The peak-caller Model-based Analysis of ChIP-Seq (MACS2) algorithm captures the influence of genome complexity to evaluate the significance of enriched ChIP regions. These MACS2-binding significance scores were evaluated for *DOT1L* and *VEGF* in each individual ChIP-Seq experiment. Finally, BigWig data of HIF1A ChIP-Seq preformed in several cell types were visualized around the *DOT1L* TSS. All the data were mapped to the reference human genome (hg19) using the Integrative Genomics Viewer. Our analyses included the following publicly available ChIP-Atlas data sets: SRX2584127, SRX4741788, SRX4741791, SRX4802362, SRX4802347, SRX4108918, SRX3583255, SRX3583256, SRX3342251, SRX3342252, SRX4096728, SRX4096729, SRX666556, SRX1034772, SRX1995010, SRX3051209, SRX4802363, SRX4802349 and SRX4096727.

### Luciferase reporter assay.

The full human *DOT1L* promoter sequence (–1000 bp to +91 bp relative to TSS) and a shorter promoter sequence (–412 bp to +91 bp relative to TSS), in which the conserved overlapping tandem HREs were removed, were amplified by PCR and cloned into the pGL3-Basic luciferase reporter vector (Promega, E1751) using the KpnI and XhoI sites (promoter sequence defined using the EPD [https://epd.epfl.ch//index.php]). Primers used for the amplification are described in Supplemental Table 3. C28/I2 cells were seeded in 24-well plates. After 24 hours, the cells were transfected with the full or shorter promoter reporter plasmids using Lipofectamine LTX Reagent with PLUS Reagent (Invitrogen) according to the manufacturer’s protocol. After 24 hours, the cells were stimulated with vehicle (DMSO) or IOX2 (20 μM) for 72 hours. The luciferase activity was assessed with Luciferase Assay System (Promega). As a control, the total protein concentration was determined by the Pierce BCA Protein Assay Kit (Thermo Fisher Scientific). Finally, the ratio of Firefly luciferase to total protein was determined as relative luciferase activity.

### Immunofluorescence.

C28/I2 cells were seeded in Nunc Lab-Tek II (Thermo Fisher Scientific) chamber slides. The following day, the cells were treated with 20 μM IOX2, 50 μM IOX2, or vehicle DMSO for 72 hours. Then, the cells were fixed using 3.7% formaldehyde in PBS for 10 minutes and antigen retrieval was performed using 1% SDS in PBS for 2 minutes. The cells were blocked in 1% BSA for 30 minutes and incubated with primary antibody against H3K79me2 (Abcam, ab3594, 1:1000) for 1 hour. Next, the cells were incubated for 1 hour with Alexa Fluor 555–conjugated secondary antibody (Thermo Fisher Scientific, A-31572, 1:1000) and DAPI (Thermo Fisher Scientific, 62249, 1:10,000). Images were taken using an Olympus IX83 microscope. Fluorescence quantification was performed with ImageJ Software (NIH Image) using 20 images per condition for each independent experiment.

### DMM mouse model of OA.

Wild-type male C57BL/6 mice were purchased from Janvier. At 8 weeks of age, posttraumatic OA was induced by DMM. To this end, a mild instability of the knee was obtained by surgical transection of the medial menisco-tibial ligament of the right knee (32). Sham surgery served as control. The knees were histologically analyzed 12 weeks after surgery.

### Intra-articular IOX2 injections.

One week after DMM surgery, mice were intra-articularly injected with IOX2 (0.5 mg/kg) or vehicle (30% PEG400 in PBS) every 10 days for a total of 7 injections. Twelve weeks after surgery, the knees were harvested and analyzed.

### Histology.

Dissected mouse knees were fixed overnight at 4°C in 2% formaldehyde, decalcified for 3 weeks in 0.5 M EDTA pH 7.5, and embedded in paraffin. All staining was performed on 5 μm thick sections. Severity of disease was determined by histological scores on hematoxylin-safraninO–stained sections throughout the knee (6 sections at 100 μm distance). Cartilage damage and synovitis were assessed based on OARSI guidelines (64). Osteophytes were scored following an in-house scoring system earlier reported (9). Images were taken using a Visitron Systems microscope (Leica Microsystems GmbH).

### Immunohistochemistry.

Immunohistochemistry was performed on 5 μm thick paraffin-embedded EDTA-decalcified knee sections. Heat induced epitope retrieval was performed using a Citrate-EDTA buffer (pH 6.2) for 10 minutes at 95°C. Sections were treated with 3% H_2_O_2_/methanol for 10 minutes to inactivate endogenous peroxidase, blocked in goat serum for 30 minutes, and incubated overnight at 4°C with primary antibodies against DOT1L (Abcam, ab64077, 6 μg/ml) or HIF1A (Abcam, ab82832, 10 μg/ml) or for 90 minutes with primary antibody against H3K79me2 (Abcam, ab3594, 1 μg/ml). Rabbit IgG (Santa Cruz, sc-2027) was used as negative control. Avidin-biotin complex amplification (Vectastain ABC kit, Vector Laboratories) was used, except for the immunohistochemical detection of H3K79me2. Peroxidase goat anti-rabbit IgG (Jackson Immunoresearch) was applied for 30 minutes, and peroxidase activity was determined using DAB. Images were taken using an Olympus IX83 microscope. Quantification of the DAB staining was performed with a color deconvolution plugin (Jacqui Ross, Auckland University) in ImageJ Software (NIH Image). Quantification was performed using the average of 2 technical replicates for 5 different mice per condition, with staining intensity reported relative to the average of the 5 SHAM+Vehicle mice.

### Micromasses.

Primary hACs were cultured in 10 μl droplets (micromasses) in 24-well plates at a density of 300,000 cells/micromass. Culture medium was changed twice per week and consisted of DMEM/F12 (Gibco), 10% FBS (Gibco), 1% (vol/vol) antibiotic/antimycotic (Gibco), 1% L-glutamine (Gibco) and Insulin-Transferrin-Selenium (ITS) (Thermo Fisher Scientific). Micromasses were treated with vehicle (DMSO) or DOT1L inhibitor EPZ (10 μM) (Chemietek) under normoxic (21% O_2_) or hypoxic (1% O_2_) conditions for 2 weeks. The micromasses were washed with PBS and fixed with ice-cold methanol for 1 hour at –20°C. After rinsing with PBS, the micromasses were stained with Alcian Blue (0.1% AB 8GX, MilliporeSigma) for 2.5 hours, washed with water, and air dried. Quantification of the staining was performed by dissolving the micromasses with 6 M guanidine (MilliporeSigma) for 6 hours and measuring the absorbance at 595 nm with a spectrophotometer (BioTek Synergy).

### Statistics.

Data analysis and graphical presentation were performed with GraphPad Prism version 8 and R-Studio Version 1.1.463 (packages *car*, *coin*, *emmeans*, *ggpubr*, *lme4*, *lmerTest*, *MuMIn*, *nlme*, *piecewiseSEM*, *readr*, *rstatix,* and *tidyverse*). Data are presented as mean ± SEM and as individual data points, representing the mean of technical replicates, as indicated in the figure legends. Gene expression data and ratio data were log-transformed for statistical analysis. All tests performed were 2 tailed where applicable. For comparisons against a hypothetical mean in the ChIP experiments, 1-sample, 2-tailed *t* test was used. For comparisons between 2 groups, unpaired 2-tailed Student’s *t* test with Welch correction in case of unequal variance was used. For comparisons between 2 groups with nonindependent data, paired 2-tailed Student’s *t* test was used. For comparisons between more than 2 groups, 1-way ANOVA was used. For dose-response experiments, generalized least square regression models were used for the cell line experiments, and mixed models were used for primary hAC experiments, with individual donor as random factor. Two-way ANOVA was used to study interactions and main effects between independent categorical variables. Holm-Bonferroni and Šidák corrections were used for multiple comparisons. Data are reported by F values and *t* values, with degrees of freedom and exact *P* values (if *P* > 0.0001) in Supplemental Table 4 where applicable. *P* values of pair-wise comparisons are indicated in the graphs. Effect sizes (*R*^2^ or pseudo-*R*^2^ for regression models or differences between means in 2 group comparisons) are reported in Results section. Distribution of the dependent variables was assessed by histogram inspection. Model assumptions were further checked by QQ plots and homoscedasticity plots. Homogeneity of variance was evaluated by standardized residuals versus fit plot. For some generalized least square models, the regression was better fitted using the constant plus power variance function structure (Supplemental Table 4). For analysis of the animal experiment, the nonparametric Kruskal-Wallis rank-sum test was used followed by pairwise Holm-Bonferroni–corrected Wilcoxon tests. Effect size *r* and 95% CIs were calculated with 500 bootstrap replications. *P* values of less than 0.05 were considered significant.

### Study approval.

Primary hACs were isolated from patients undergoing hip replacement surgery for osteoporotic or malignancy-associated fractures, with informed consent and ethical approval of the University Hospitals Leuven Ethics Committee and Biobank Committee (Leuven, Belgium) (S56271). According to Belgian law and UZ Leuven’s biobank policies, the hip joints are considered human biological residual material. Only the age and sex of the patients were shared between the surgeons and the investigators involved in this study. The material is fully anonymized without links to medical files. All mouse model studies were performed with approval from the Ethics Committee for Animal Research (P114-2008, P198-2012, P159-2016; KU Leuven) (license no. LA1210189).

## Author contributions

ADR, SM, and RJL planned the study and designed all the in vitro, ex vivo, and in vivo experiments. ADR and AEN performed in vitro and ex vivo experiments. FMFC performed the animal experiments. CC contributed to experimental design. FC cloned and provided the plasmid constructs. RJL and ADR are responsible for all statistical analyses. LCF and AS provided essential materials. ADR, SM, and RJL wrote the manuscript.

## Supplementary Material

Supplemental data

## Version 1 11/02/2021

In-Press Preview

## Version 2 12/22/2021

Electronic publication

## References

[1] Xia B (2014). Osteoarthritis pathogenesis: a review of molecular mechanisms. Calcif Tissue Int.

[2] Bijlsma JW (2011). Osteoarthritis: an update with relevance for clinical practice. Lancet.

[3] Feng Q (2002). Methylation of H3-lysine 79 is mediated by a new family of HMTases without a SET domain. Curr Biol.

[4] Nguyen AT, Zhang Y (2011). The diverse functions of Dot1 and H3K79 methylation. Genes Dev.

[5] Steger DJ (2008). DOT1L/KMT4 recruitment and H3K79 methylation are ubiquitously coupled with gene transcription in mammalian cells. Mol Cell Biol.

[6] Castaño Betancourt MC (2012). Genome-wide association and functional studies identify the DOT1L gene to be involved in cartilage thickness and hip osteoarthritis. Proc Natl Acad Sci U S A.

[7] Castaño-Betancourt MC (2016). Novel genetic variants for cartilage thickness and hip osteoarthritis. PLoS Genet.

[8] Monteagudo S (2017). DOT1L safeguards cartilage homeostasis and protects against osteoarthritis. Nat Commun.

[9] Cornelis FMF (2019). Increased susceptibility to develop spontaneous and post-traumatic osteoarthritis in Dot1l-deficient mice. Osteoarthritis Cartilage.

[10] Lories RJ (2013). To Wnt or not to Wnt: the bone and joint health dilemma. Nat Rev Rheumatol.

[11] Zhu M (2009). Activation of beta-catenin signaling in articular chondrocytes leads to osteoarthritis-like phenotype in adult beta-catenin conditional activation mice. J Bone Miner Res.

[12] Dreos R (2015). The Eukaryotic Promoter Database: expansion of EPDnew and new promoter analysis tools. Nucleic Acids Res.

[13] Livyatan I (2015). BindDB: An integrated database and webtool platform for “reverse-ChIP” epigenomic analysis. Cell Stem Cell.

[14] Messeguer X (2002). PROMO: detection of known transcription regulatory elements using species-tailored searches. Bioinformatics.

[15] Sandelin A (2004). ConSite: web-based prediction of regulatory elements using cross-species comparison. Nucleic Acids Res.

[16] Ghosh D (2000). Object-oriented transcription factors database (ooTFD). Nucleic Acids Res.

[17] Matsuura K (2012). Histone H3K79 methyltransferase Dot1L is directly activated by thyroid hormone receptor during Xenopus metamorphosis. Cell Biosci.

[18] Szklarczyk D (2019). STRING v11: protein-protein association networks with increased coverage, supporting functional discovery in genome-wide experimental data sets. Nucleic Acids Res.

[19] Greene CS (2015). Understanding multicellular function and disease with human tissue-specific networks. Nat Genet.

[20] Choudhry H, Harris AL (2018). Advances in hypoxia-inducible factor biology. Cell Metab.

[21] Schipani E (2001). Hypoxia in cartilage: HIF-1alpha is essential for chondrocyte growth arrest and survival. Genes Dev.

[22] Okada K (2020). Hypoxia-inducible factor-1 alpha maintains mouse articular cartilage through suppression of NF-κB signaling. Sci Rep.

[23] Bouaziz W (2016). Interaction of HIF1α and β-catenin inhibits matrix metalloproteinase 13 expression and prevents cartilage damage in mice. Proc Natl Acad Sci U S A.

[24] Majmundar AJ (2010). Hypoxia-inducible factors and the response to hypoxic stress. Mol Cell.

[25] Fernández-Torres J (2017). Hypoxia-inducible factors (HIFs) in the articular cartilage: a systematic review. Eur Rev Med Pharmacol Sci.

[26] Fernández-Torres J (2017). Role of HIF-1α signaling pathway in osteoarthritis: a systematic review. Rev Bras Reumatol Engl Ed.

[27] Yang S (2010). Hypoxia-inducible factor-2alpha is a catabolic regulator of osteoarthritic cartilage destruction. Nat Med.

[28] Ryu JH (2011). Interleukin-6 plays an essential role in hypoxia-inducible factor 2α-induced experimental osteoarthritic cartilage destruction in mice. Arthritis Rheum.

[29] Fukasawa M (2004). Identification and characterization of the hypoxia-responsive element of the human placental 6-phosphofructo-2-kinase/fructose-2,6-bisphosphatase gene. J Biochem.

[30] Finger F (2003). Molecular phenotyping of human chondrocyte cell lines T/C-28a2, T/C-28a4, and C-28/I2. Arthritis Rheum.

[31] Oki S (2018). ChIP-Atlas: a data-mining suite powered by full integration of public ChIP-seq data. EMBO Rep.

[32] Glasson SS (2007). The surgical destabilization of the medial meniscus (DMM) model of osteoarthritis in the 129/SvEv mouse. Osteoarthritis Cartilage.

[33] Lotz MK, Kraus VB (2010). New developments in osteoarthritis. Posttraumatic osteoarthritis: pathogenesis and pharmacological treatment options. Arthritis Res Ther.

[34] Kim W (2014). The histone methyltransferase Dot1/DOT1L as a critical regulator of the cell cycle. Cell Cycle.

[35] Nguyen AT (2011). DOT1L regulates dystrophin expression and is critical for cardiac function. Genes Dev.

[36] Wasserman WW, Sandelin A (2004). Applied bioinformatics for the identification of regulatory elements. Nat Rev Genet.

[37] Thoms BL (2013). Hypoxia promotes the production and inhibits the destruction of human articular cartilage. Arthritis Rheum.

[38] Schrobback K (2012). Effects of oxygen and culture system on in vitro propagation and redifferentiation of osteoarthritic human articular chondrocytes. Cell Tissue Res.

[39] Markway BD (2013). Hypoxia promotes redifferentiation and suppresses markers of hypertrophy and degeneration in both healthy and osteoarthritic chondrocytes. Arthritis Res Ther.

[40] Duval E (2012). Molecular mechanism of hypoxia-induced chondrogenesis and its application in in vivo cartilage tissue engineering. Biomaterials.

[41] Okada Y (2005). hDOT1L links histone methylation to leukemogenesis. Cell.

[42] Jones B (2008). The histone H3K79 methyltransferase Dot1L is essential for mammalian development and heterochromatin structure. PLoS Genet.

[43] Gelse K (2008). Role of hypoxia-inducible factor 1 alpha in the integrity of articular cartilage in murine knee joints. Arthritis Res Ther.

[44] Hu S (2020). Stabilization of HIF-1α alleviates osteoarthritis via enhancing mitophagy. Cell Death Dis.

[45] Myllyharju J (2003). Prolyl 4-hydroxylases, the key enzymes of collagen biosynthesis. Matrix Biol.

[46] Leung IK (2010). Structural and mechanistic studies on γ-butyrobetaine hydroxylase. Chem Biol.

[47] Walport LJ (2012). Mechanisms of human histone and nucleic acid demethylases. Curr Opin Chem Biol.

[48] Salminen A (2015). 2-Oxoglutarate-dependent dioxygenases are sensors of energy metabolism, oxygen availability, and iron homeostasis: potential role in the regulation of aging process. Cell Mol Life Sci.

[49] Harrison AP, Pierzynowski SG (2008). Biological effects of 2-oxoglutarate with particular emphasis on the regulation of protein, mineral and lipid absorption/metabolism, muscle performance, kidney function, bone formation and cancerogenesis, all viewed from a healthy ageing perspective state of the art--review article. J Physiol Pharmacol.

[50] van Leeuwen F (2002). Dot1p modulates silencing in yeast by methylation of the nucleosome core. Cell.

[51] Shanower GA (2005). Characterization of the grappa gene, the Drosophila histone H3 lysine 79 methyltransferase. Genetics.

[52] Woo Park J (2015). RE-IIBP methylates H3K79 and induces MEIS1-mediated apoptosis via H2BK120 ubiquitination by RNF20. Sci Rep.

[53] Shah YM, Xie L (2014). Hypoxia-inducible factors link iron homeostasis and erythropoiesis. Gastroenterology.

[54] Feng Y (2010). Early mammalian erythropoiesis requires the Dot1L methyltransferase. Blood.

[55] Chen X (2020). Methyltransferase Dot1l preferentially promotes innate IL-6 and IFN-β production by mediating H3K79me2/3 methylation in macrophages. Cell Mol Immunol.

[56] Eltzschig HK, Carmeliet P (2011). Hypoxia and inflammation. N Engl J Med.

[57] Brown E, Taylor CT (2018). Hypoxia-sensitive pathways in intestinal inflammation. J Physiol.

[58] Kwesi-Maliepaard EM (2020). The histone methyltransferase DOT1L prevents antigen-independent differentiation and safeguards epigenetic identity of CD8^+^ T cells. Proc Natl Acad Sci U S A.

[59] Zhang L (2020). Loss of histone H3 K79 methyltransferase Dot1l facilitates kidney fibrosis by upregulating Endothelin 1 through histone deacetylase 2. J Am Soc Nephrol.

[60] Shu S (2019). Hypoxia and hypoxia-inducible factors in kidney injury and repair. Cells.

[61] Jain RK (2009). A new target for tumor therapy. N Engl J Med.

[62] Wang X (2019). Depletion of H3K79 methyltransferase Dot1L promotes cell invasion and cancer stem-like cell property in ovarian cancer. Am J Transl Res.

[63] Vlaming H, van Leeuwen F (2016). The upstreams and downstreams of H3K79 methylation by DOT1L. Chromosoma.

[64] Glasson SS (2010). The OARSI histopathology initiative — recommendations for histological assessments of osteoarthritis in the mouse. Osteoarthritis Cartilage.

