# Supplementary material for: Hypoxia induces DOT1L in articular cartilage to protect against osteoarthritis
Source: JCI Insight. 2021 Dec 22;6(24):e150451. doi: 10.1172/jci.insight.150451 (PMC8783684; doi:10.1172/jci.insight.150451)
Supplement: Supplemental data [file jciinsight-6-150451-s124.pdf]

SUPPLEMENTAL INFORMATION

Supplemental Figure 1

A

>DOT1L; human; range -1000bp to 100bp.

TACCTCAGCCCCCGCAGTAGCTGGGATTACAGGCGCCCGCCACCGCGCCCAGCTAATTTTTGTATTTTTA
ATAGAGACAGGGTTTCACCATGTTGGCCAGGCTGATCTCGAACTCCTGACCTCAGGTGATCTTCCCGCCT
CGGCCTCACAAAGTGCTGGGATTACAGGCATGAGCCAGGGCTCCTGGCCGTGAGAGGGGGTTTCTCCATG
TTGGTCAGGCTGGTCTCGAACTCCTGACCTCAGGTGATCTGCCCGCCTTGGCCTCCCAAAGTGCTGGGAT
TACAGGGGTGAATCACCGCGCCTGGCCTTTTTTTTTTTTTTTGTACCCAATAAACAGCATTGTTGTCGAA
TGAATACTGGACTTGGAGCTACAGCAAGTCCTGGACCGTGACTCTTATGGGGGAAATTCGGATTTTTGG
TTTTACTAAGCCGTGTGTGGGGAGGTGTCCGGCGTCCTCCTCCTGGGACGGGATTCGAACCCGCTATCCG
ACGGGCCCGCCCACAGGGTCTCCCCGGGTCCCCGCTTCGGCCGGCGAGTGGGGGAAGGGGTCGGCCGAG
GGCAACCGAGGACGTGCGTGCGTACGTTCGTGCGTGCGTGGATTCGGGCGGGCGGGCGAGTCCACGGGGC
GGGGCGCCGAGGGGGTGGCGCGCGCGGGTCGGCCCGCTGGGCGGCGGGCACGCGCCGGCGTCTTCGCTCC
GGGCTCCCCTAGCGCGCGGGGCGAGTGGTTCCGCCCGGCCCCCGGCTCATTGTGCTCGCTTCACGCCGGC
CCAAGATGGCGGAGGCGCTGGAGGCCCCGGGCCTGTGACTACAAAGAGGGAGTCGGGGGCCGGGCCGGAC
CGGAGCGCGGCGGCGGCGGCGGCGGCGGCCGAGGCCGAGGCCAGGCCCCCTCCCCTCAGCCTCCCGCCCC
TCCCTCCCGCCCGCCCTCCTCCGCCCACCGGCGGCCCCGCCCCTCCCCCAACCGCCCGCCTAGCATGGTG
CGGCGGCCGCGCGCGCGGACATGGGGGAGAAGCTGGAGCTGAGACTGAAGTCGCCCGTGGGGGCTGAGCC
CGCCGTCTACCCGTGGCCGCTGCCGGTCTACGTGAGTGCCGCCCTCCACCG

B

>DOT1L; mouse; range -1000bp to 100bp.

TTCTAGCTCCGGATCCTTCTTTTGGGGGCTGGCAAGCGCTAGGATGAGGGGTGGGAGCCATCTCCCCTAG
ATTACATTCACTCCCATGTAGTCAGGAAGAAGAAGCAGCTATCCAGAAGCGGCTGGTGAGCTGGTGACAG
CTCACCTCAAGGTAACTTATTCTCAGAAGGTTTTGGGGGGAGGGAACTTAAGTATTAGAAGGATTGGAAT
GGCCTAAGAAGGAAATAAGTTTGGCTCCTGACTCTGTAAAGTTCTGGGCAGGTCAGAGAATAACATTGCT
TTGAACTCTTAATTGAGAGCCTCCGACCTATAGGGTCCAATTTGACTGATGATTTATTAATTAACTTATT
GCTCACATCTTTAATCCCAGCTCTCAGGAGGCAGAGGCAGGTGGATTTTGGTAAATTCGAGGTCAGACTG
GTCAACAGAATGAGTTCCTGGACAGCCAGGGACACCCTGTCTCAAAAAAAAAAAAAAAAAAAAAAAGCATG
TAAATAAACAATAAGCAGATAGGCTAGAAAAGTGAAAAAATAGAAAAGCTAAAGAAGGGAAAAAATTGAA
AAATGAAAAAAAAAAGAAAGAAAGAAAAAGCCAGAAAAACAGAAAAAGAAAATTGACGGGAAGGGAAGAG
AAAGAAAAAGAACAGGAAATGGAAAGAAAAGAAAAAAGCAAAGCAAAGCGAAGGGACGCGAAGCAAGCCT
GTGCAACTGGGATTTGAACTTGACCCCGCCTGGTCTCTCTCGCTTTCCTCCGCAGGGTTTCCCCGGGTCC
CCGCTTCCGGCTGGCGGCGACGTCGGAGGGCAACCGAGGACGTGCGTGCGCCGCTGCGTGCGTAAGTGCG
TGCGTCGGTCGGAGGAGGGCGAGTCCAAGGGGGCGTGGCGTGCGAGGGGTGGCGCGCGCGGCGCGGCGGG
CGGAGGCTCTGGGCGCGCACCGCCTCTTGGCTCCCGGCCTCCCCCGCGCGCGCTCGCCGAGTGGTTCCGC
CCGACCCCCCGGCTCATTGTGCCTTCTCCTCACGCCGGCCCAAGATGGCGGCGGCTCTAGACGCCCCGGC
TCTGTGACTCCTACAAAGAGGGGAGCTGGGGCCACACGGGAGCGGCGGCCG

C

>DOT1L; rat; range -1000bp to 100bp.

ATGTATGTTTATGCACCACGATTGCCTGGTGCATGGAGAGGCCAGAAGAGGGCGTTGGAACCCTGGGAAT
TCAGTCAGAGATGGTTGTGAGCCCGAGGGAGGGCTGGGAATGGAATCTAAGGTTCTCTAAAAGAGCAGCC
TGTAGTCTTAGCTACTGAACCATCTCTCTAACCCATTTGATGATTTTGTTTTTCAGTTTTGTTCTTGCTG
TTAGGAATGGTTTCACTCTGTAGCTCAAGTGGGCTTTGAGTCGTGATCCCTTGCCTCAGCTTCCCAAGAC
CTTGGATTATGGGTCATAGATTCACGGTTTTGTTAGTGTGAAACTGCATAGAAAGTCGGAAAATATCTTC
AAAAAGAATGAAAAGAAGCTAGGCGTGGTGGTGCACACCTTTAATCCCAGCACTAGGGAGGGAGGCAGAG
GCGGGGAATCTTGGTGAATTTGAGGTAAGACTGGTCAACAGAGTAGACGGAGTTCCAGGACAGCCAGGGC
TACCGTGTCTCAAAAAAAAAAAAAAAACCAAATAAGTAAATAAACAATAAATAAGTGGATAAGATAAAAA
AAAGTGAAAAGAGAAACAGAAAAAGGAAAAAAGCCGGAAAAACAGAAAAAGAATAGGGACGAGAAGAGAA
AGAAAAGGAAAAGGAAAGGAAAGAAAAGAAAAAGAAAAGAAAAGAAAAAAAAGCAAAGCAAAGCGAGCCT
GCGCAACTGGGACTTGAACTTGTCCCCACCTGGTCTCTCTCGCTTGCCTCAGCAGGGTTTCCCCGGGTCC
CCGCTTCCGGCTGGCGGTGGCGTCGGAGGGCAACCGAGGACGTGCGTGCGACCCTGCGTGCGAACGTGCG
TGCGTCGGCGGGAGGTGGGCGAGTCCAAGGGGGCGTGGCGTACGAGGGGTGGTGCGCGCGGCGCGGCGGG
CGGAGGCTTTGGGCGCGCACCGCCTCTTGGCTCCCGGCCTCCCCCGCGCGCGCGCGCCCAGTGGTTCAGC
CCGACCCCCCGGCTCATTGTGCCTTCTCCTCACGCCGGCCCAAGATGGCGGCGGCGCTAGACGCCCCGGC
TCTGTGACTCCTACAAAGAGGGGAGCTGGGGCCACACGGGAGCGGTGGCCG

Supplemental Figure 1 Predicted hypoxia response elements (HREs) in human, mouse and rat *DOT1L* gene promoters. (A-B-C) Sequences of the human (A), mouse (B) and rat (C) *DOT1L* gene promoters from 1000 base pairs (bp) upstream of the transcription start site (TSS) to 100 bp downstream of the TSS as determined by the Eukaryotic Promoter Database. HREs with core consensus sequence 5'-(A/G)CGTG-3', potential binding sites for HIF heterodimers, are marked with a yellow box. The bp downstream of the TSS are highlighted in grey.

Supplemental Figure 2

Multiple sequence alignment by MUSCLE

```
human -------------TACCTC--------AGCCCCGCAGTAGCTGGGATTACAGGCGCCCGCCACCGCGCCCAG-------CTAATTTTTGTATTTTT
mouse TTCTAGCTCCGGATCCTTCTTTTG---GGGGCTGGCAAGCGCTAGGATGAGGGGTGGGAGCCATCTCCCCTAGATTACATTCACTCCCATGTAGTC
rat   ATGTATGTTTATGCACCACGATTGCCTGGTGCATGGAGAGGCCAGAAGAGGGCGTTGGAA-------CCCTGG---GAATTCAGTCAGAGATGGTT
                     *  *         *  *  * *   **  * *      *             **  *        * * *      *  *

human AATA----GAGACAGGGTTT-----------------------------CACCATGTTGGCCAGGCTG-------ATCTC---------GAACTCC
mouse AGGAAGAAGAAGCAG-----------------------CTATCCAGAAGCGGCTGGTG-----AGCTGGTGA--CAGCTCACCTCAAGGTAACTTA
rat   GTGAGCCCGAGGGAGGGCTGGGAATGGAATCTAAGGTTCTCTAAAAGAGCAGCCTGTAGTCTTAGCTACTGAACCATCTCTC-------TAACCCA
         *    **   **                                  *  *  **       ***        * ***          ***

human TGACCTCAGGTGATCT-------------------------------------------------------------------------------T
mouse T--TCTCAGAAGGTTTTGGGGGGAGGGAACTTAAGTATT-------------AGAAGGATTGGA----ATGGCCTAAGAAGGAAATAAGTTTGGCT
rat   T--TT---GATGATTTTGTT---------TTTCAGTTTTGTTCTTGCTGTTAGGAATGGTTTCACTCTGTAGCTCAAGTGGGCTTTGAGTCGTGAT
      *       *  * * *                                                                               *

human CC--CGCCTCGGCCTCACAAAGTGCTGGGATTACAGG-----------CATGAGCCAGGGCTCCTGGCCGTGAG----------AGGGGGTT----
mouse CC--TGACTCTG-----TAAAGTTCTGGG----CAGGTCAGAGAATAACATTGCTTTGAACTCTTAATTGAGAGCCTCCGACCTATAGGGTC----
rat   CCCTTGCCTCAGCTTC-CCAAGACCTTGGATTATGGGTCATAGATT--CACGGTTTTG------TTAGTGTGAAACT---GCATAGAAAGTCGGAA
      **   * *** *       ***  ** **      **           **       *      *    * **           *    **

human ----TCTCCATGT-----------------------TGGTCAGGCTGGTCTCGAACTCCTGACCT-----------------CAGGTGATCTGCCC
mouse --------CAATTTGACTGATGATTTATTAATTAACTTATTGCTCACATCTTTAATCCCAGCTCT----CAGGAGGCAGAGGCAGGTGG-------
rat   AATATCTTCAAAAAGAATGAAAAGAAGCTAGGCG--TGGTGGTGCACACCTTTAATCCCAGCACTAGGGAGGGAGGCAGAGGCGGG-GA-------
               **                         *  *    *    **   **  **  * **                 * ** *

human GCCTTGGCCTCCCAAAGTGCTGGGATTACAGGGGT--------------GAATCACCG---CGCCTGGCCTTTTTTTTTTTT--------------
mouse ATTTTGG-----TAAATT--CGAGGTCAGACTGGTCAACAGAAT-----GAGTTCCTGGACAGCCAGGGACACCCTGTCTCAAAAAAAAAAAAAAA
rat   ATCTTGG-----TGAATT--TGAGGTAAGACTGGTCAACAGAGTAGACGGAGTTCCAGGACAGCCAGGGCTACCGTGTCTCAAAAAAAAAAAAAAA
         ****       ** *   *   * * * *  ***              ** *  * *    *** **       * * *

human --------TTTGTACCCAATAAACAGCA----------------TTGTTGTCGAATGAATA--CTGGACTTGGAGCTACAGCAAGTCCTGGACCGT
mouse AAAAAAAGCATGTA---AATAAACAATAAGCAGATAGGCTAGAAAAGTGAAAAAATAGAAAAGCTAAAGAAGGGGAAAAATTGAAAAATGAAAAAA
rat   ACCAAA--TAAGTA---AATAAACAATA---------------------AATAAGTGGATAAGATAAA-------AAAAAGTGAA-----AAGAGA
         ***   ********  *       *                         * *  * *   *  *         * *   *       *

human GACTCTTATGGGGGGAAATTCGGATTTTTGGTTTTACTAAGCCGTGTGTGGGGAGGTG--------------------------------------
mouse AAAAGAAAGAAAGAAAAAGCCAGAAAAACAG----AAAAAGAAAATTGACGGGAAGGGAAGAGAAAGAAAAAGAACAGGAAATGGAAAGAAAAGAA
rat   AACAGAAAAAGGAAAAAAGCCGGAAAAACAG----AAAAAGAATAGGGACGAGAAGAGAAAGAAAAGGAAAAGGAAAGGAAA--GAAAAGAAAAAG
       *     *       ***  * **       *    *   ***     *  * ** * *

human -----------------------TCCGGCGTCCTCCTCCTGGGACGGGATTCGAACCCGCTATCCGACGGGCC-----------CGCCCACAGGGT
mouse AAAAGCAAAGCAAAGCGAAGGGACGCGAAGCAAGCCTGTGCAACTGGGATTTGAACTTGAC-CCCGCCTGGTCTCTCTCGCTTTCCTCCGCAGGGT
rat   AAAAGAAAAG-AAAAAAAAGCAAAGCAAAGCGAGCCTGCGCAACTGGGACTTGAACTTGTC-CCCACCTGGTCTCTCTCGCTTGCCTCAGCAGGGT
                               *   *    ***        **** * ****   *    **  * ** *           *  *  ******

human CTCCCCGGGTCCCCGCTTCGGGCCGGCGAGTGGGGAAGGGGTCGGCCGAGGGCAACCGAGGACGTGCGTGCG----TACGTTCGTGCGTGCGTGG
mouse TTCCCCGGGTCCCCGCTTCCGGCTGGC-----------GGCGACGTCGGAGGGCAACCGAGGACGTGCGTGCGCCGCTGCGTGCGTAAGTGCGTGC
rat   TTCCCCGGGTCCCCGCTTCCGGCTGGC-----------GGTGGCGTCGGAGGGCAACCGAGGACGTGCGTGCGACCCTGCGTGCGAACGTGCGTGC
       ****************** *** ***           ** * ** * *************************    * *** **   *******

human ATTCG-GGCGGGCGGGCGAGTCCAC-GGGGCGGGGCGCCGAGGGGGTGGCGCGCGCGGGTCGGCCCGCTG-GGCGGCGGGCACGCGCCGGCGTCTT
mouse GTCGGTCGGAGGAGGGCGAGTCCAAGGGGGCGTGGCGTGCGAGGGGTGGCGCGCGCGGCGCGGCGGGCGGAGGCTCTGGGCGCGCACC-GCCTCTT
rat   GTCGGCGGGAGGTGGGCGAGTCCAAGGGGGCGTGGCGTACGAGGGGTGGTGCGCGCGGCGCGGCGGGCGGAGGCTTTGGGCGCGCACC-GCCTCTT
       *  *  *  ** ***********  ****** ****     ******* ********  ****  ** * ***   **** *** ** ** ****

human CGCTCCGGGCTCCCCTAGCGCGCG--GGGCGAGTGGTTCCGCCCGG-CCCCCGGCTCATTGTGC--TCGCTTCACGCCGGCCCAAGATGGCGGAGG
mouse GGCTCCCGGCCTCCCCCGCGCGCGCTCGCCGAGTGGTTCCGCCCGACCCCCCGGCTCATTGTGCCTTCTCCTCACGCCGGCCCAAGATGGCGGCGG
rat   GGCTCCCGGCCTCCCCCGCGCGCGCGCGCCCAGTGGTTCAGCCCGACCCCCCGGCTCATTGTGCCTTCTCCTCACGCCGGCCCAAGATGGCGGCGG
       ***** ***  ***  *******   * * ******** *****  *****************  ** * ********************** **

human CGCTGGAGGCCCCGGGCCTGTGA---CTACAAAGAGGGAGTCGGGGGCCGGGCCGGACCGGAGCGCGGCGGCGGCGGCGGCGGCGGCCGAGGCCGA
mouse CTCTAGACGCCCCGGCTCTGTGACTCCTACAAAGAGGGGAGCTGGGGCCACACGGGA---------------------------------------
rat   CGCTAGACGCCCCGGCTCTGTGACTCCTACAAAGAGGGGAGCTGGGGCCACACGGGA---------------------------------------
      * ** ** *******  ******   ************   * ******   * ***

human GGCCAGGCCCCCTCCCCTCAGCCTCCCGCCCCTCCCTCCCGCCCGCCCTCCTCCGCCCACCGGCGGCCCCGCCCCTCCCCCAACCGCCCGCCTAGC
mouse ------------------------------------------------------------------------------------------------
rat   ------------------------------------------------------------------------------------------------

human ATGGTGCGGCGGCCGCGCGCGCGGACATGGGGGAGAAGCTGGAGCTGAGACTGAAGTCGCCCGTGGGGGCTGAGCCCGCCGTCTACCCGTGGCCGC
mouse -----GCGGCGGCCG---------------------------------------------------------------------------------
rat   -----GCGGTGGCCG---------------------------------------------------------------------------------
           **** *****

human TGCCGGTCTACGTGAGTGCCGCCCTCCACCG
mouse -------------------------------
rat   -------------------------------
```

Supplemental Figure 2 Alignment of the human, mouse and rat *DOT1L* gene promoters. Alignment of the human, mouse and rat sequences of the *DOT1L* gene promoters from 1000 base pairs (bp) upstream of the transcription start site (TSS) to 100 bp downstream of the TSS as determined by the Eukaryotic Promoter Database. This alignment was performed using MUSCLE. Hypoxia response elements (HREs) are marked with a yellow box.

Supplemental Figure 3

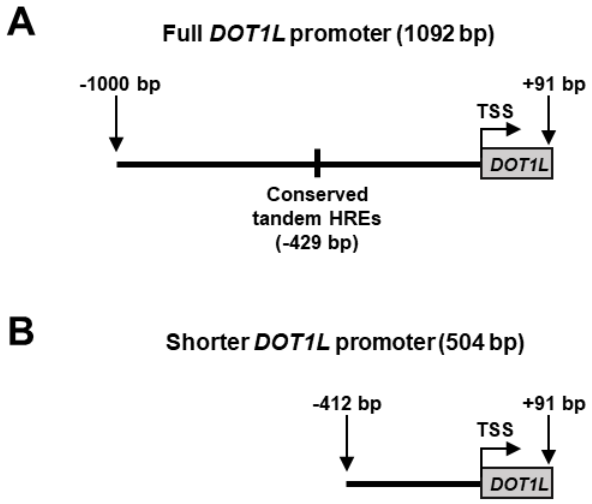

Supplemental Figure 3 Promoter constructs for the luciferase reporter assay. (A) The full human *DOT1L* promoter from 1000 base pairs (bp) upstream to 91 bp downstream relative to the transcription start site (TSS) as defined by the Eukaryotic Promoter Database was cloned into the pGL3 basic luciferase reporter vector. This 1092 bp fragment contains the conserved overlapping tandem Hypoxia response elements (HREs) located at -429 bp relative to the TSS. (B) The shorter *DOT1L* promoter from 412 bp upstream to 91 bp downstream relative to the TSS was cloned into the pGL3 basic luciferase reporter vector. This 504 bp fragment does not contain the conserved overlapping tandem HREs.

Supplemental Figure 4

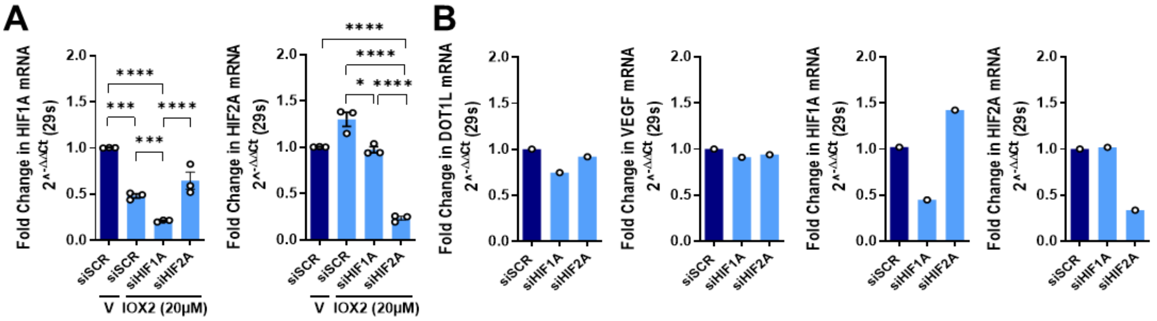

Supplemental Figure 4 Efficiency of siRNA-mediated gene silencing in C28/I2 cells. (A) Real-time PCR analysis of *HIF1A* and *HIF2A* in human articular chondrocyte C28/I2 cells after treatment with hypoxia mimetic IOX2 (20 µM) and siRNA-mediated silencing of *HIF1A* (siHIF1A), *HIF2A* (siHIF2A) or scrambled control (siSCR) (n=3, **p*<0.05, ****p* <0.001, *****p*<0.0001 Sidak-corrected for 6 tests in one-way ANOVA). (B) Real-time PCR analysis of *DOT1L*, *VEGF*, *HIF1A* and *HIF2A* in C28/I2 cells after siRNA-mediated silencing of *HIF1A*, *HIF2A* or scrambled control (siSCR) (n=1). Bar graphs are mean±sem.

Supplemental Figure 5

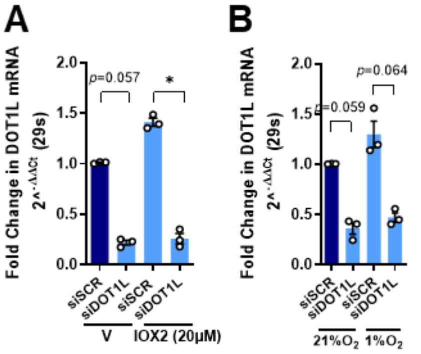

Supplemental Figure 5 Efficiency of siRNA-mediated gene silencing in hACs. (A-B) Real-time PCR analysis of *DOT1L* in primary human articular chondrocytes (hACs) after treatment with hypoxia mimetic IOX2 (20 µM) or vehicle (V) (A) (n=3, *p<0.05, Sidak-corrected for 6 tests in two-way ANOVA) or culturing in hypoxic conditions (1% O_2) (B) and siRNA-mediated silencing of *DOT1L* or scrambled control (siSCR) (n=3, Sidak-corrected for 6 tests in two-way ANOVA). Bar graphs are mean±sem.

Supplemental Figure 6

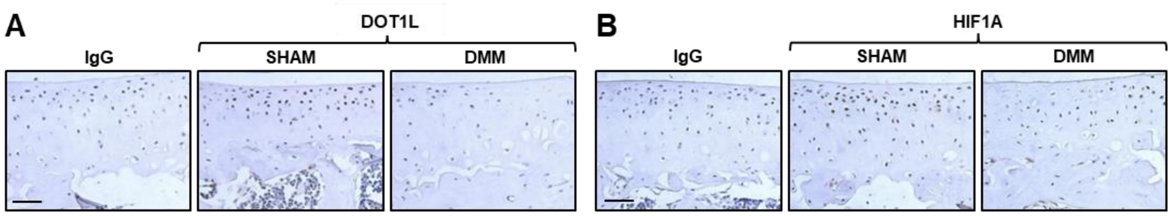

Supplemental Figure 6 DOT1L and HIF-1A protein levels in osteoarthritis murine articular cartilage. (A-B) Immunohistochemical detection of DOT1L (A) and HIF1A (B) in the articular cartilage of wild-type mice with OA triggered by destabilisation of the medial meniscus (DMM) surgery compared to sham operated mice. The images are representative of three different animals. Scale bar, 50 µm.

Supplemental Table 1

Human primers used for qPCR

Primer name	Sequence
**hACAN_fw**	ATCCGAGACACCAACGAGAC
**hACAN_rv**	CACTCATTGGCTGCTTCCTG
**hCOL2A1_fw**	TGGCAGAGATGGAGAACCTG
**hCOL2A1_rv**	CATCAAATCCTCCAGCCATC
**hDOT1L_fw**	GGATCTCAAGCTCGCTATGG
**hDOT1L_rv**	GTCGATGGCACGGTTGTACT
**hHIF1A_fw**	TCATCCATGTGACCATGAGG
**hHIF1A_rv**	TTCCTCGGCTAGTTAGGGTACA
**hHIF2A_fw**	CTGCGACCATGAGGAGATTC
**hHIF2A_rv**	GTACGGCCTCTGTTGGTGAC
**hS29_fw**	GGGTCACCAGCAGCTGTACT
**hS29_rv**	AAACACTGGCGGCACATATT
**hTCF1_fw**	CCCCCAACTCTCTCTCTACGA
**hTCF1_rv**	TGCCTGAGGTCAGGGAGTAG
**hVEGF_fw**	TGCAGATTATGCGGATCAAACC
**hVEGF_rv**	TGCATTCACATTTGTTGTGCTGTAG

Supplemental Table 2

Human primers used for ChIP-qPCR

Primer name	Sequence
**hDOT1Lprom_fw**	CCAATAAACAGCATTGTTGTCG
**hDOT1Lprom_rv**	CCCACACACGGCTTAGTAAAA
**hVEGFprom_fw**	TCACTTTCCTGCTCCCTCCT
**hVEGFprom_rv**	GCAATGAAGGGGAAGCTCGA

Supplemental Table 3

Primers used for the PCR amplification for luciferase assay

Construct	Primer	Sequence
**Full DOT1L promoter (1092 bp)**	Forward	GGTACCTACCTCAGCCCCCGCAGTA
	Reverse	ACGCTCGAGGCGGCACTCACGTAGACC
**Shorter DOT1L promoter (504 bp)**	Forward	GGTACCCGTGCGTGCGTGGATTCG
	Reverse	ACGCTCGAGGCGGCACTCACGTAGACC

Supplemental Table 4

Statistical Analysis Details

**FIGURE 3A**	Generalized least square model with constant plus power variance function structure
DOT1L	beta=0.006, t=6.209 p<0.0001
VEGF	beta=0.018, t=10.65 p<0.0001

**FIGURE 3B**	Generalized least square model with constant plus power variance function structure
DOT1L	beta=0.015, t=11.14 p<0.0001
VEGF	beta=0.005, t=24.62 p<0.0001

**FIGURE 3C**	Welch-correct unpaired t-test
DOT1L	t_2=4.940 p=0.0385
VEGF	t_2=6.231 p=0.0248

**FIGURE 3F**	Generalized least square model
Immuno-fluorescent	beta=0.007, t=17.32 p<0.0001

**FIGURE 4A**	Two-way ANOVA	V:Empty vs. V:DOT1L	V:Empty vs. IOX2:Empty	V:Empty vs. IOX2:DOT1L	V:DOT1L vs. IOX2:Empty	V:DOT1Lvs. IOX2:DOT1L	IOX2:Empty vs. IOX2:DOT1L
Luciferase activity	$F_{1,8}$ = 21.71 p=0.0016 for IOX2 treatment<br>$F_{1,8}$ = 21.86 p=0.0016 for reporter<br>$F_{1,8}$ = 1.38 p=0.755 for interaction	t_8= 3.078 p=0.088	t_8= 3.067 p=0.089	t_8= 6.601 p=0.001	t_8= 0.0115 p=0.99	t_8= 3.522 p=0.046	t_8= 3.534 p=0.045

**FIGURE 4B**	One-way ANOVA	siSCRv vs. siSCR	siSCRv vs. siHIF1A	siSCRv vs. siHIF2A	siSCR vs. siHIF1A	siSCR vs. siHIF2A	siHIF1A vs. siHIF2A
DOT1L	$F_{3,8}$ = 36.28	t_8=9.53 p<0.0001	t_8=4.426 p=0.0132	t_8=8.10 p=0.0002	t_8=5.10 p=0.0055	t_8=1.427 p=0.7207	t_8=3.678 p=0.0369
VEGF	$F_{3,8}$ = 113.0	t_8=17.25 p<0.0001	t_8=10.68 p<0.0001	t_8=14.2 p<0.0001	t_8=6.57 p=0.0011	t_8=3.042 p=0.0923	t_8=3.526 p=0.0458

**FIGURE 5A**	Linear mixed model
DOT1L	beta=0.01, t=2.73 p=0.046
VEGF	beta=0.023, t=15.71, p<0.0001

**FIGURE 5B**	Paired t-test
DOT1L	t_2=3.280 p=0.0817
VEGF	t_2=10.27 p=0.0093

**FIGURE 5C**	Linear mixed model
COL2	beta=0.02, t=4.27, p=0.0009
ACAN	beta=0.024, t=4.69, p=0.0007

**FIGURE 5D**	Paired t-test
COL2	t_2=14.51 p=0.0047
ACAN	t_2=16.84 p=0.0035

**FIGURE 5E**	Two-way ANOVA	Vehicle-siSCR vs vehicle-siDOT1L	Vehicle-siSCR vs IOX2-siSCR	Vehicle-siSCR vs IOX2-siDOT1L	Vehicle-siDOT1L v IOX2-siSCR	Vehicle-siDOT1L vs IOX2-siDOT1L	IOX2-siSCR vs IOX2-siDOT1L
COL2	$F_{1,2}$= 3.378 p=0.2075for IOX2 treatment<br>$F_{1,2}$= 9.659 p=0.089 for DOT1L silencing<br>$F_{1,2}$= 3.33 p=0.2084 for interaction	t_2=2.254 p=0.6306	t_2=6.480 p=0.1303	t_2=1.644 p=0.8103	t_2=8.734 p=0.0747	t_2=3.898 p=0.310	t_2=4.836 p=0.218
ACAN	$F_{1,2}$= 0.768 p=0.4732 for IOX2 treatment<br>$F_{1,2}$= 25.93 p=0.0365 for DOT1L silencing<br>$F_{1,2}$= 1.14 p=0.3977 for interaction	t_2=2.792 p=0.496	t_2=1.343 p=0.8933	t_2=2.958 p=0.461	t_2=4.136 p=0.282	t_2=0.1656 p=0.9	t_2=4.301 p=0.265
TCF1	$F_{1,2}$= 31.30 p=0.0305 for IOX2 treatment<br>$F_{1,2}$= 80.53 p=0.0122 for DOT1L silencing<br>$F_{1,2}$= 8.81 p=0.097 for interaction	t_2=4.880 p=0.215	t_2=3.384 p=0.383	t_2=5.694 p=0.164	t_2=8.265 p=0.083	t_2=0.814 p=0.985	t_2=9.079 p=0.069

**FIGURE 5F**	Two-way ANOVA	Normoxia-siSCR vs normoxia-siDOT1L	Normoxia-siSCR vs hypoxia-siSCR	Normoxia-siSCR vs hypoxia-siDOT1L	Normoxia- siDOT1 vs hypoxia-siSCR	Normoxia-siDOT1L vs hypoxia-siDOT1L	Hypoxia-siSCR vs hypoxia-siDOT1L
COL2	$F_{1,2}$= 226.3 p=0.0044 for oxygen exposure	t_2=3.435 p=0.375	t_2=12.68 p=0.0364	t_2=9.61 p=0.0623	t_2=16.1 p=0.0227	t_2=13.04 p=0.0344	t_2=3.07 p=0.4383

	$F_{1,2}$= 12.95 *p*=0.069 for DOT1L silencing $F_{1,2}$= 0.07 *p*=0.812 for interaction						
ACAN	$F_{1,2}$= 158.4 *p*=0.0063 for oxygen exposure $F_{1,2}$= 65.75 *p*=0.0149 for DOT1L silencing $F_{1,2}$= 0.012 *p*=0.9225 for interaction	t_2=11.0 *p*=0.0476	t_2=15.58 *p*=0.0243	t_2=4.39 *p*=0.2555	t_2=26.6 *p*=0.0083	t_2=15.43 *p*=0.0248	t_2=11.18 *p*=0.0456
TCF1	$F_{1,2}$= 2.609 *p*=0.2476 for oxygen exposure $F_{1,2}$= 130.6 *p*=0.0076 for DOT1L silencing $F_{1,2}$= 9.23 *p*=0.093 for interaction	t_2=5.945 *p*=0.152	t_2=3.553 *p*=0.357	t_2=6.691 *p*=0.123	t_2=9.50 *p*=0.0637	t_2=0.745 *p*=0.989	t_2=10.24 *p*=0.055
**FIGURE 5G**	Two-way ANOVA	Normoxia V vs. normoxia EPZ	Normoxia V vs hypoxia V	Normoxia-V vs hypoxia EPZ	Normoxia UPZ vs hypoxia V	Normoxia EPZ vs hypoxia EPZ	Hypoxia V vs hypoxia EPZ
	$F_{1,2}$= 77.07 *p*=0.0127 for oxygen exposure $F_{1,2}$= 45.62 *p*=0.0212 for EPZ treatment $F_{1,2}$= 16.08 *p*=0.057 for interaction	t_2=11.7 *p*=0.0426	t_2=14.55 *p*=0.0278	t_2=8.528 *p*=0.078	t_2=26.2 *p*=0.0087	t_2=20.22 *p*=0.0145	t_2=6.022 *p*=0.1487
**FIGURE 6B**	Kruskal -Wallis	SHAM V vs DMM		DMM V vs DMM IOX2		SHAM V vs DMM IOX2	
OARSI	χ^2=14.297 *p*=0.0008	Z=3.09 *p*=0.002		Z=2.31 *p*=0.021		Z=2.41 *p*=0.016	
**FIGURE 6C**	Kruskal -Wallis	SHAM V vs DMM V		DMM V vs DMM IOX2		SHAM V vs DMM IOX2	
osteophyte	χ^2=15.36 *p*=0.0004	Z=2.71 *p*=0.0068		Z=2.22 *p*=0.0267		Z=2.71 *p*=0.0068	
**FIGURE 6D**	Kruskal -Wallis	SHAM V vs DMM		DMM V vs DMM IOX2		SHAM V vs DMM IOX2	
synovitis	χ^2=10.93 *p*=0.0064	Z=2.57 *p*=0.01		Z=1.75 *p*=0.08		Z=1.13 *p*=0.26	
**FIGURE 6E**	One-way ANOVA	SHAM V vs DMM		DMM V vs DMM IOX2		SHAM V vs DMM IOX2	
HIF1A	$F_{2,12}$=45.45 *p*<0.0001	t_{12}=9.533 *p*<0.0001		t_{12}=4.909 *p*=0.001		t_{12}=4.624 *p*=0.0018	
DOT1L	$F_{2,12}$=15.07 *p*=0.0005	t_{12}=4.574 *p*=0.0019		t_{12}=4.917 *p*=0.001		t_{12}=0.3435 *p*=0.98	
H3K79	$F_{2,12}$=45.12 *p*<0.0001	t_{12}=7.792 *p*<0.0001		t_{12}=8.60 *p*<0.0001		t_{12}=0.808 *p*=0.82	

**FIGURE S4A**	One-way ANOVA	Vehicle-siSCR vs IOX2-siSCR	Vehicle-siSCR vs IOX2-siHIF1a	Vehicle-siSCR vs IOX2-siHIF2a	IOX2-siSCR vs IOX2-siHIF1a	IOX2-siSCR vs IOX2-siHIF2a	IOX2-siHIF1a vs IOX2-siHIF2a
HIF1A	$F_{3,8}$ = 77.26	t_8=7.013 p=0.0007 P<0.0001	t_8=14.80 p<0.0001	t_8=4.374 p=0.0141	t_8=7.786 p=0.0003	t_8=2.639 p=0.1659	t_8=10.42 p<0.0001
HIF2A	$F_{3,8}$ = 191.4	t_8=3.208 p=0.0725 P<0.0001	t_8=0.4094 p=0.9992	t_8=18.36 p<0.0001	t_8=3.617 p=0.0402	t_8=21.57 p<0.0001	t_8=17.96 p<0.0001
**FIGURE S5A**	Two-way ANOVA	Vehicle-siSCR vs vehicle-siDOT1L	Vehicle-siSCR vs IOX2-siSCR	Vehicle-siSCR vs IOX2-siDOT1L	Vehicle-siDOT1L vs IOX2-siSCR	Vehicle-siDOT1L vs IOX2-siDOT1L	IOX2-siSCR vs IOX2-siDOT1L
DOT1L	$F_{1,2}$= 7.730 p=0.1087 for IOX2 treatment $F_{1,2}$= 140.9 p=0.0070 for DOT1L silencing $F_{1,2}$= 0.7453 p=0.4789 for interaction	t_2=9.986 p=0.0578	t_2=2.150 p=0.6601	t_2=9.057 p=0.0697	t_2=12.14 p=0.0397	t_2=0.9286 p=0.9727	t_2=11.21 p=0.0463
**FIGURE S5B**	Two-way ANOVA	Normoxia-siSCR vs normoxia-siDOT1L	Normoxia-siSCR vs hypoxia-siSCR	Normoxia-siSCR vs hypoxia-siDOT1L	Normoxia-siDOT1L vs hypoxia-siSCR	Normoxia-siDOT1L vs hypoxia-siDOT1L	Hypoxia-siSCR vs hypoxia-siDOT1L
DOT1L	$F_{1,2}$= 12.32 p=0.0725 for oxygen exposure $F_{1,2}$= 91.27 p=0.0108 for DOT1L silencing $F_{1,2}$= 0.06641 p=0.8207 for interaction	t_2=9.809 p=0.0599	t_2=2.292 p=0.6201	t_2=7.153 p=0.1087	t_2=12.10 p=0.0399	t_2=2.656 p=0.5270	t_2=9.445 p=0.0644
